# Supplementary material for: Multi-omics identify falling LRRC15 as a COVID-19 severity marker and persistent pro-thrombotic signals in convalescence
Source: Nat Commun. 2022 Dec 15;13:7775. doi: 10.1038/s41467-022-35454-4 (PMC9753891; doi:10.1038/s41467-022-35454-4)
Supplement: Supplementary file 1 — Supplementary Information [file 41467_2022_35454_MOESM1_ESM.pdf]

Supplementary Material for 'Multi-omics identify falling LRRC15 as a COVID-19 severity marker and persistent pro-thrombotic signals in convalescence' by Gisby *et al*.

Table of Contents

-Supplementary Text

-Supplementary Figures

-Supplementary Tables

-Titles for Supplementary Data

-Supplementary References

Supplementary Text

Sensitivity analyses exploring the effects of including additional clinical covariates

We considered whether clinical factors related to ESKD might affect the PBMC transcriptome or the plasma proteome, and potentially confound our differential expression or severity analyses. We first evaluated whether cause of ESKD, diabetes or duration of time since first commencing haemodialysis had any impact on PCA plots for the transcriptome or the proteome. These covariates did not have any visible effect on the PCA plots, in either COVID-19 negative or COVID-19 positive ESKD patients (**Supplementary Figure 24**).

Nevertheless, to ensure that our results were not being affected by confounding, we next performed sensitivity analyses comparing our primary statistical model to a model with additional covariates (adjusting for cause of ESKD, diabetes status and duration of time spent on dialysis). We performed these sensitivity analyses for the Wave 1 differential gene expression model (COVID-19 positive versus negative ESKD patients), the differential protein abundance model, and for the transcriptomic and proteomic analyses testing for association with clinical severity. We found that adjustment for these covariates had very little effect on the comparison of interests (**Supplementary Figure 2**).

The Wave 2 differential gene expression and protein abundance models compared COVID-19 positive samples to pre-infection samples from the same individuals, and accordingly inter-individual variation is inherently controlled for.

How do COVID-19 related transcriptomic and proteomic signatures in ESKD patients relate to those seen in non-ESKD patient studies?

Transcriptomics

To address this question, we compared our RNA-seq results to the COvid-19 Multi-omics Blood ATlas (COMBAT) Consortium study [1], which is also from a United Kingdom healthcare setting. The COMBAT study performed whole blood bulk RNA-seq from 77 individuals with COVID-19 and 10 healthy controls (https://doi.org/10.5281/zenodo.6120249). We re-analysed these RNA-seq data using the same normalisation and modelling methods that we used for our data so that they were as comparable as possible to the results of our study. Specifically, we used linear mixed models to test the association of genes with COVID-19 status (positive vs. negative) and clinical severity (encoded as an ordinal variable; mild, severe, critical). We also tested the enrichment of gene sets for these comparisons using the GSVA method that we used in the analysis of our own data.

We then compared the effect estimates in our cohorts to those from our re-analysis of the COMBAT data. For the comparison of COVID-19 positive versus negative samples, we found a high degree of concordance between our study and the COMBAT study, both for the gene-level differential expression analysis (COMBAT versus our Wave 1 data Pearson correlation coefficient (r) = 0.68; COMBAT versus our Wave 2 data also 0.68) and for the GSVA analysis (COMBAT versus Wave 1 r = 0.54; COMBAT versus Wave 2 r = 0.66) (**Supplementary Figure 17**). Our results for the association of gene expression with COVID-19 severity were also generally consistent with the COMBAT data. For gene-level analysis, r was 0.56 and 0.59 for Wave 1 and Wave 2, respectively. For GSVA gene sets, r was 0.44 and 0.37 for Wave 1 and 2, respectively (**Supplementary Figure 18**). The overlap in the number of significant (1% FDR) genes and gene sets between our datasets and the COMBAT data is shown in **Supplementary Figure 25.**

Given that the COMBAT RNA-seq data were generated on whole blood (versus PBMC in our study) and considering potential sources of non-biological variation between studies, these levels of inter-study concordance suggest that the transcriptomic signatures related to both COVID-19 (versus negative controls) and to COVID-19 severity are similar in the ESKD and non-ESKD patients.

Proteomics

We then compared our plasma proteomic data to that of Filbin *et al.* [2], which also used the SomaScan proteomic platform and collected samples at multiple timepoints during acute COVID-19 (https://doi.org/10.17632/nf853r8xsj.2). We additionally applied inverse-rank normalisation to each protein in their data before downstream analysis. COVID-19 severity was binarised into severe (patients who were intubated or died) and non-severe (non-intubated patients).

The effect estimates for the COVID-19 positive versus negative analyses in our study and the Filbin data were moderately correlated for the Wave 1 analysis (r = 0.45), but less so for Wave 2 (r = 0.28). We observed a similar pattern for the GSVA protein pathway analysis (Wave 1 r = 0.46; Wave 2 r = 0.22) (**Supplementary Figure 19**). For the analysis of proteins associated with severity, correlation of effect estimates with the Filbin data was modest for our Wave 1 analysis (r = 0.20) but stronger for our Wave 2 analysis (r = 0.55) and protein sets (Wave 1 r = 0.22; Wave 2 r = 0.39) (**Supplementary Figure 20**). The overlap in the number of significant (1% FDR) proteins and protein sets between our datasets and the Filbin data is shown in **Supplementary Figure 26**.

A renal effect identified in our pathway-level (GSVA) proteomic analysis between COVID-19 positive and negative samples was proximal tubule bicarbonate reclamation (see **Figure 2C**). We suspected this related to reduction in any limited remaining intrinsic renal function in ESKD patients experiencing COVID-19 and was likely a consequence rather than a driver of host inflammatory response to infection. Comparison with the data of Filbin *et al.* revealed that the key proteins in this pathway term were also significant in their data with consistent directions of effect to our data. Thus, this effect of COVID-19 on the kidneys does not appear to be ESKD-specific.

To get a broad sense of pathways that appeared distinct in our data, we next searched for GSVA protein sets that were significant (FDR < 0.01) in our ESKD patient datasets, but without any trend towards significance (even at unadjusted P > 0.05) in the Filbin *et al.* data. For the comparison of COVID-19 positive vs. negative patients, pathways significant only in our data included those related to neutrophil degranulation, oxidative stress (e.g. oxidative damage), FGFR signaling (e.g. FGFR1 ligand binding and activation, FRS mediated FGFR3 signaling), interleukin pathways (e.g. IL6 pathway, IL18 signaling pathway, interleukin 12 family signaling), metabolism (e.g. cholesterol biosynthesis, pyruvate metabolism, insulin glucose pathway) and cell death (e.g. intrinsic pathway for apoptosis) (**Supplementary Data 1U**). We highlight that endoplasmic reticulum stress

response in coronavirus infection was also among the terms that was specific to our ESKD dataset; this is clearly not an ESKD-specific enrichment, highlighting the limitations of such inter-study comparisons. For the severity comparison, the distinct pathways were all related to transcription (e.g. transport of mature transcript to cytoplasm) and splicing (e.g. spliceosome) (**Supplementary Data 1U**).

It is plausible that these pathways represent ESKD-specific pathways activated during COVID-19 infection. However, there are number of flaws and limitations to this type of inter-study comparison. Firstly, it must be emphasised that non-significant P-values do not provide evidence for an absence of effect in the data of Filbin *et al.* The hypothesis testing provides a probability of observing the data under the null hypothesis that there is no effect. Therefore, these tests cannot confirm what they already assume. Secondly, differences in study design or technical factors (e.g. sample processing, proteomic platform) may account for variation in the results of the two studies. For example, the control group in the study of Filbin *et al.* were COVID-19 negative patients admitted with acute respiratory distress whereas our controls were COVID-19 negative ESKD patients. In addition, all the patients in the study of Filbin *et al.* were hospitalised whereas we included both outpatients and inpatients.

<u>Are the temporal cytokine profiles consistent with data from non-ESKD cohorts?</u>

Using linear mixed models including a time x clinical course interaction term, we identified 50 cytokines with different temporal profiles after COVID-19 symptom onset depending on the overall clinical course of the patients (**Figure 5**). Filbin *et al.* applied a very similar mixed modelling approach to investigate severity-specific profiles using Olink immunoassays. A subtle difference is that they encoded time as a categorical variable (0, 3 and 7 days) while we treated it as a continuous variable. Of the 50 cytokines exhibiting a significant time x clinical course interaction effect in our analysis, 32 were measured on the Olink panel used by Filbin *et al.* Of these 32 proteins, they found that 28 had a significant (FDR < 0.05) time x severity interaction term.

Filbin *et al.* also performed proteomics using the SomaScan platform v4 (we used v4.1), which measured 46/50 of the cytokines significant in our analysis. We inverse-rank normalised their SomaScan data for these cytokines and applied linear mixed models with an interaction term for time and severity. 33/46 of the cytokines replicated (at unadjusted $p < 0.05$) in this analysis. These results show that there is a high degree of concordance in terms of plasma cytokines and

receptors that have a different longitudinal profile between mild and severe patients in our ESKD cohort and a non-ESKD patient group. This suggests that severe COVID-19 is characterised by common dynamic cytokine changes across patient groups.

One exception to this was EPOR, which had a significant time x disease course interaction (p = 1.7×10^{-8}) in our data but not in that of Filbin *et al.* (p = 0.47). Erythropoietin (EPO) is a hormone produced by the kidney that promotes red cell formation. In ESKD there is loss of EPO production by the kidney, and consequently patients require exogenous administration of recombinant EPO. Visualisation of the data in our ESKD patient data revealed a decline in EPOR abundance over time in severe COVID-19 and a more stable profile in mild/moderate disease (**Supplementary Figure 16**). In contrast, we did not see this longitudinal divergence according to severity in the data of Filbin *et al.* These changes are likely to reflect the changes in erythropoietin responsiveness that accompany critical illness and sepsis in ESKD patients.

<u>Does LRRC15 have a dynamic temporal trajectory in COVID-19 patients who do not have ESKD?</u>

We next investigated whether LRRC15 is a biomarker of COVID-19 severity specifically in ESKD or is applicable to more general patient groups. We identified two external studies in non-ESKD patients that measured plasma LRRC15 and have mined their data. Su *et al.* [3] performed proteomic profiling with SomaScan in two general population cohorts. Their data indicates that LRRC15 levels are significantly associated with COVID19 outcome (Benjamini-Hochberg adjusted P-value = 10^{-10}), with lower levels associated with severe disease.

A study by Filbin *et al.* [2] found that individuals with more severe COVID-19 had a different temporal profile of plasma LRRC15 (again measured using SomaScan) compared to less severe cases. We re-analysed their data to model and visualise the interaction between time and severity for LRRC15. This revealed that, as observed in our data, LRRC15 abundance fell over time in more severe patients relative to milder cases (**Supplementary Figure 22**). These findings suggest that LRRC15 is also a biomarker of severe COVID-19 in more general patient cohorts.

Supervised learning trained and tested in separate cohorts

In our primary analysis, we trained lasso and random forests models using cross-validation performed on the combined data from the Wave 1 and Wave 2 cohorts (**Figure 6A, Supplementary Figure 21A**). This approach allowed us to maximise the training data available for modelling and create confidence intervals for the performance metrics, which was an important consideration given our relatively modest sample size. However, this also meant that we could not test the models on a completely independent cohort. Therefore, to ensure that our models of COVID-19 severity would perform well given new data, we performed a secondary analysis involving a train/test split. Specifically, we trained a second set of random forests and lasso models on the transcriptomic and proteomic datasets using cross-validation in the Wave 1 cohort alone. We were then able to test the models in the Wave 2 cohort to estimate the AUC and evaluate the ability of these models to extrapolate to truly unseen data. These scores are listed in the table below.

Algorithm	Data type	AUC
Random forests	Proteomics	0.77
Lasso	Proteomics	0.84
Random forests	RNA-seq	0.96
Lasso	RNA-seq	0.70

We observed greater variability between the performance metrics of the 4 models than those estimated in our primary analysis using cross-validation. However, it is important to stress these AUC estimates are generated from testing a single time on the Wave 2 cohort and thus are unlikely to be as accurate as the estimates from our primary analysis (cross-validation on the combined Wave 1 and 2 data) which were averaged over 200 cross-validation iterations. Nevertheless, they demonstrate that these supervised learning models can generalise to completely unseen data.

Next, we ensembled these four models, allowing each one an equally weighted vote to classify COVID-19 severity. This model performed well, with an AUC score of 0.89 in the Wave 2 cohort. The ensemble model performed better than the average of its four constituent models, potentially indicating that combining models trained on each data modality can create a more consistent classifier of severity.

Supplementary Figures

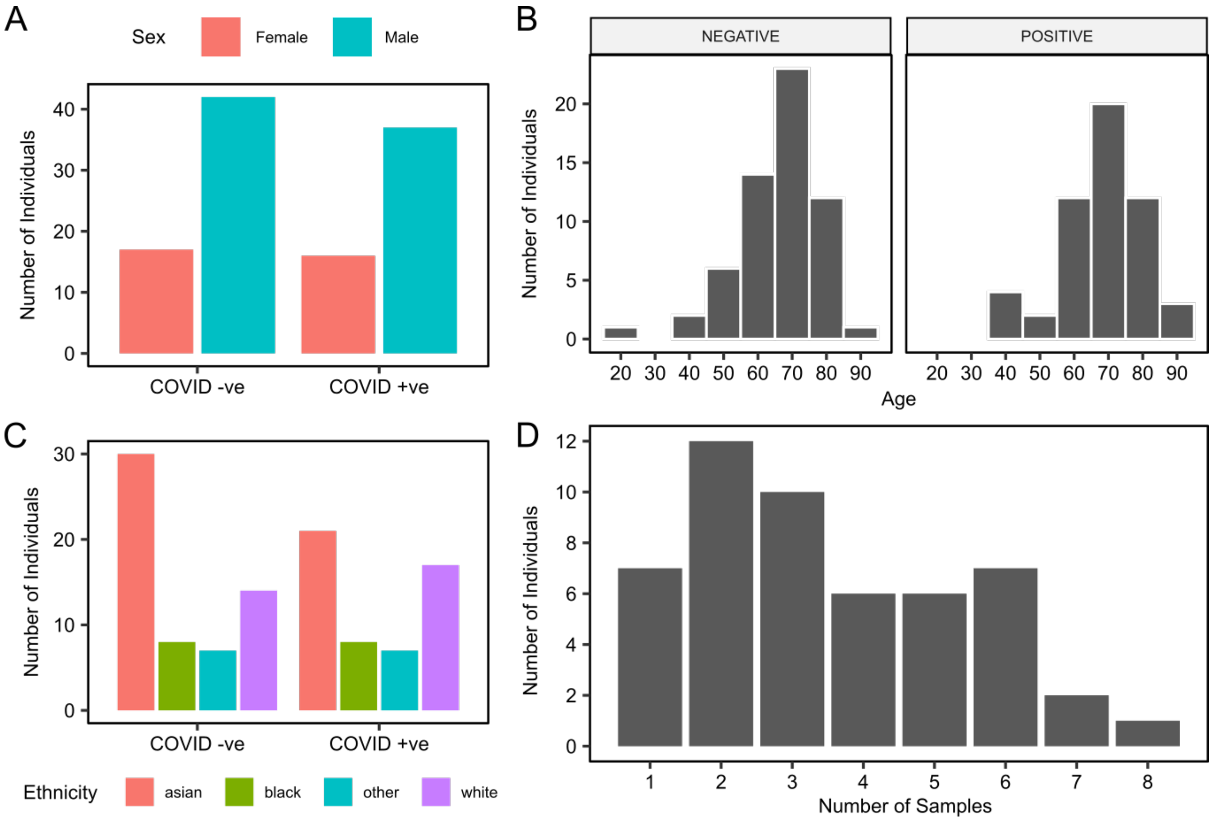

Supplementary Figure 1: Characteristics of the Wave 1 cohort. The number of COVID-19 positive and negative patients, stratified by **A**), sex **B**), age **C**) ethnicity. **D**) Number of serial PBMC samples with RNA-seq data available for COVID-19 patients.

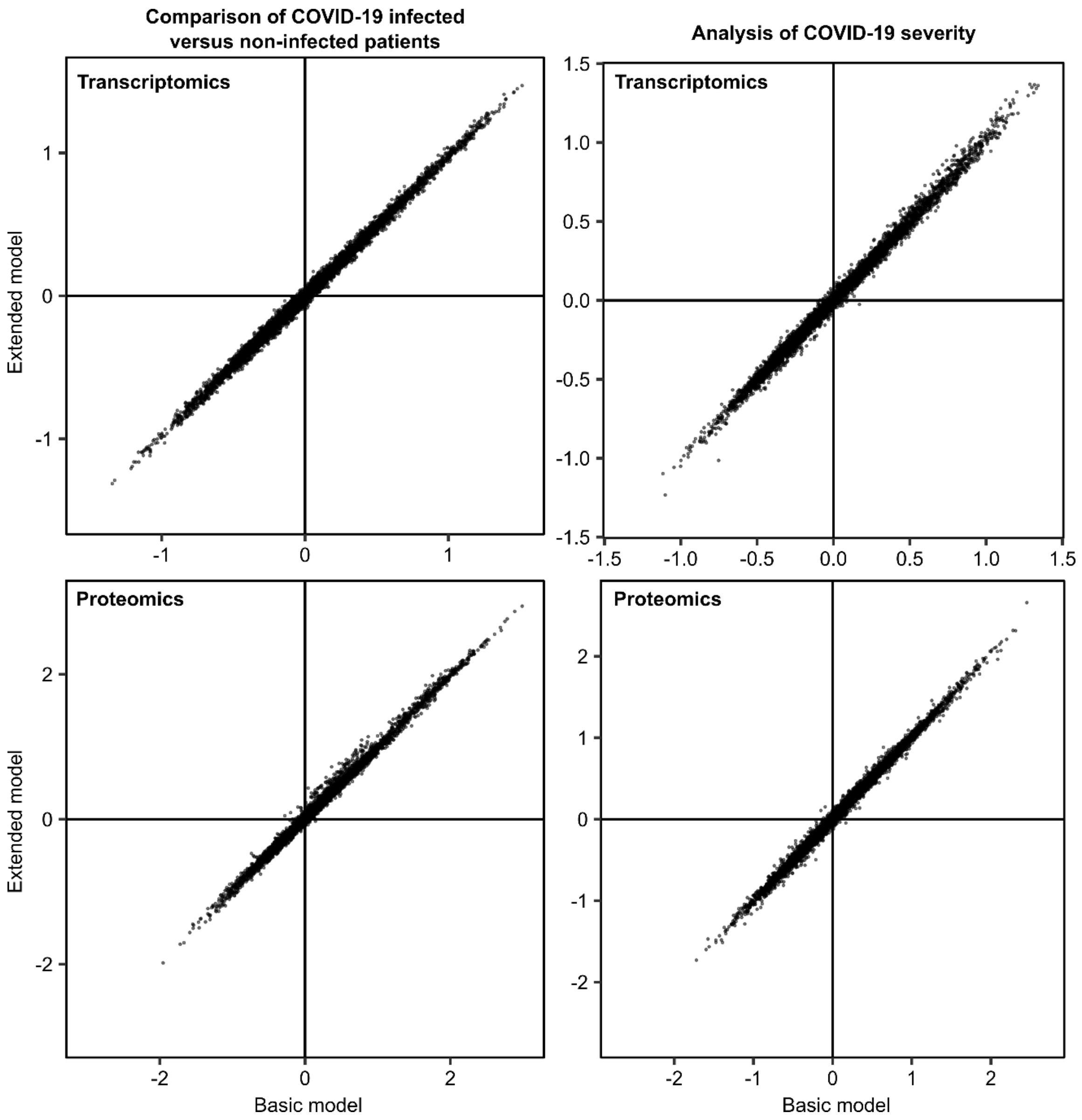

Supplementary Figure 2: The effect of including additional covariates in the Wave 1 differential gene expression and protein abundance comparisons. Each point represents a molecular feature (genes in upper panels, proteins in lower panels).The x-axis for each panel represents the LMM model estimates (betas) for the molecular feature from the model adjusted for age, sex and ethnicity. The y-axis represents the betas from an extended model with additional covariates representing cause of ESKD (diabetic nephropathy, glomerulonephritis/autoimmune, hypertension/vascular, or other/unknown), diabetes status, and time since dialysis (measured as the square root of days since the patient began dialysis).

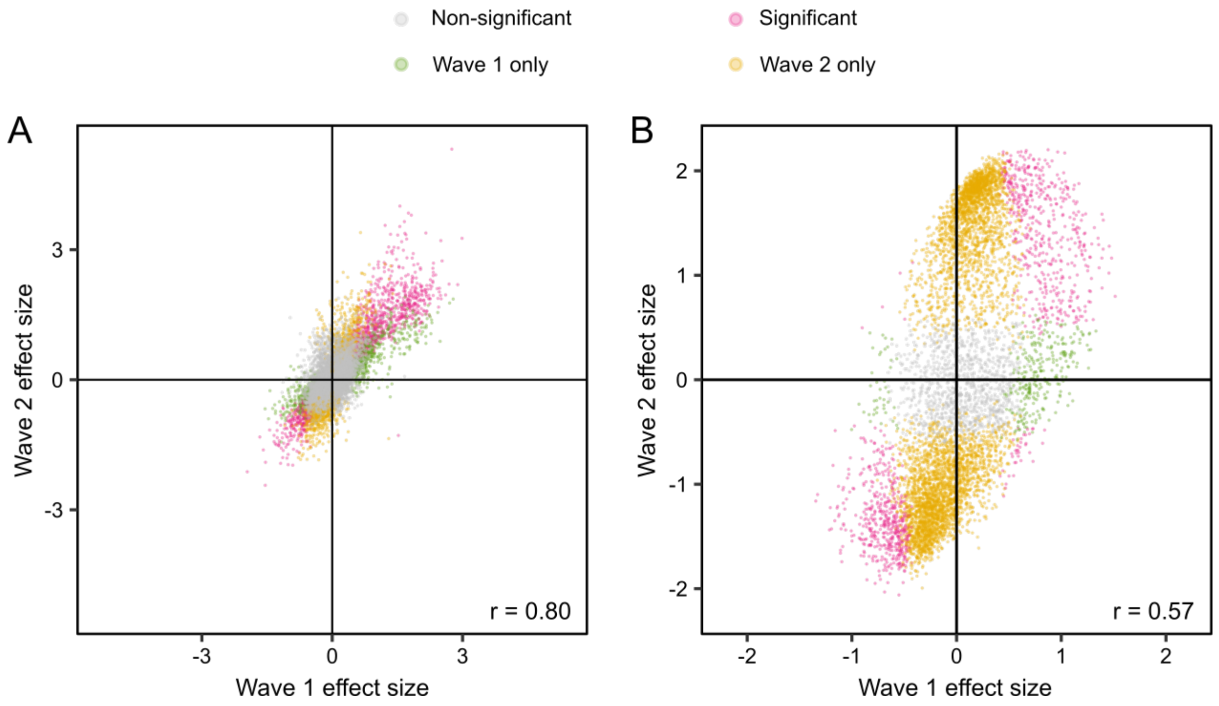

Supplementary Figure 3: A) Comparison of effect sizes (coefficients from LMM) for the Wave 1 and Wave 2 COVID-19 positive versus negative differential expression analyses for the transcriptome. Each point is a gene, coloured according to its significance in the Wave 1 and 2 analyses. r= Pearson’s correlation coefficient. **B)** As A, for the proteome.

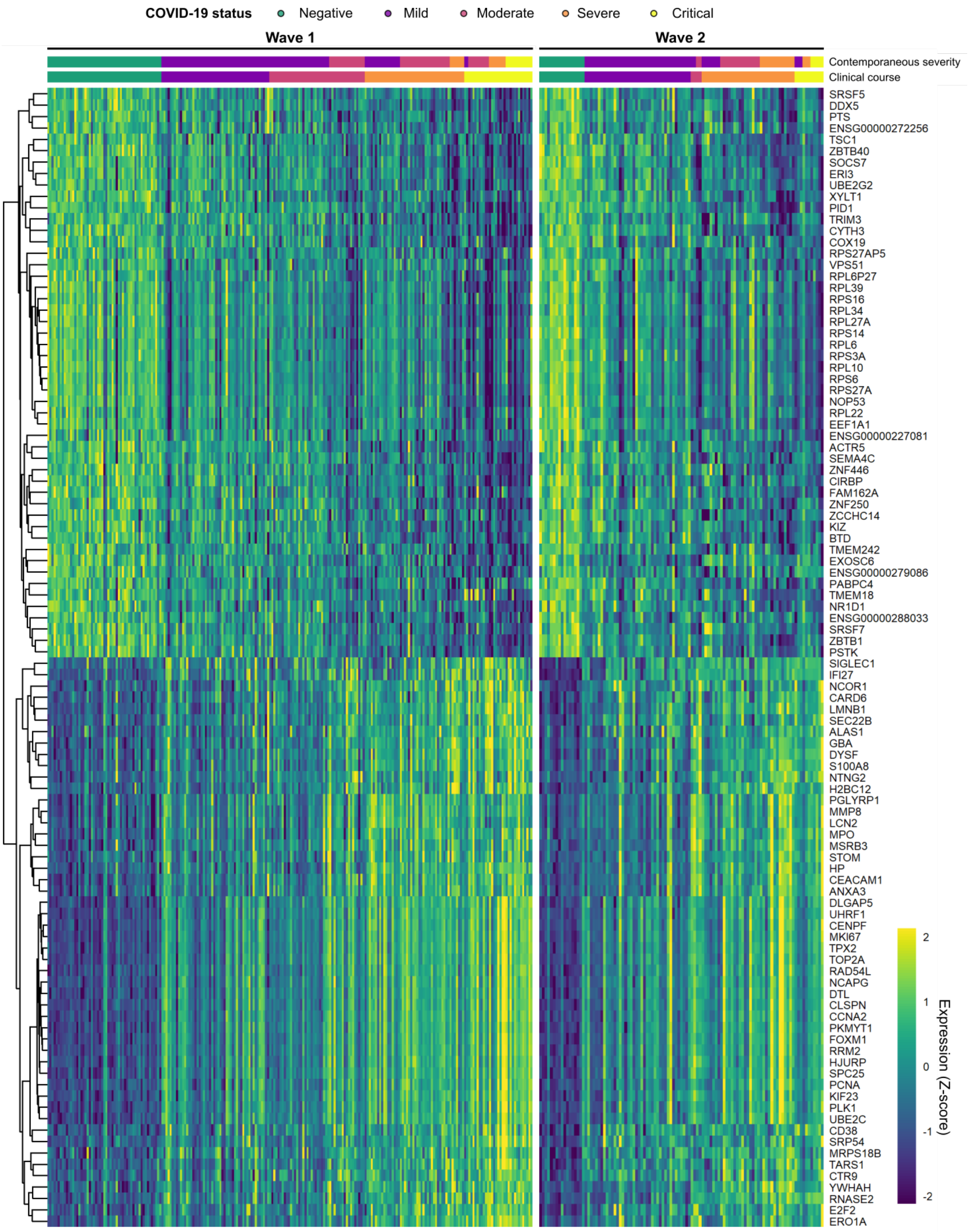

Supplementary Figure 4: Genes differentially expressed in COVID-19. Heatmap for the 100 transcriptomic features most significantly differentially expressed between COVID-19 positive and negative samples, according to robust rank aggregation (RRA). These genes were significant in both cohorts (1% FDR, LMM). Columns are ordered by COVID-19 status and severity. For the Wave 1 heatmap, genes are ordered by hierarchical clustering; the Wave 2 heatmap is ordered to match this. Each feature was scaled and centred separately in each dataset.

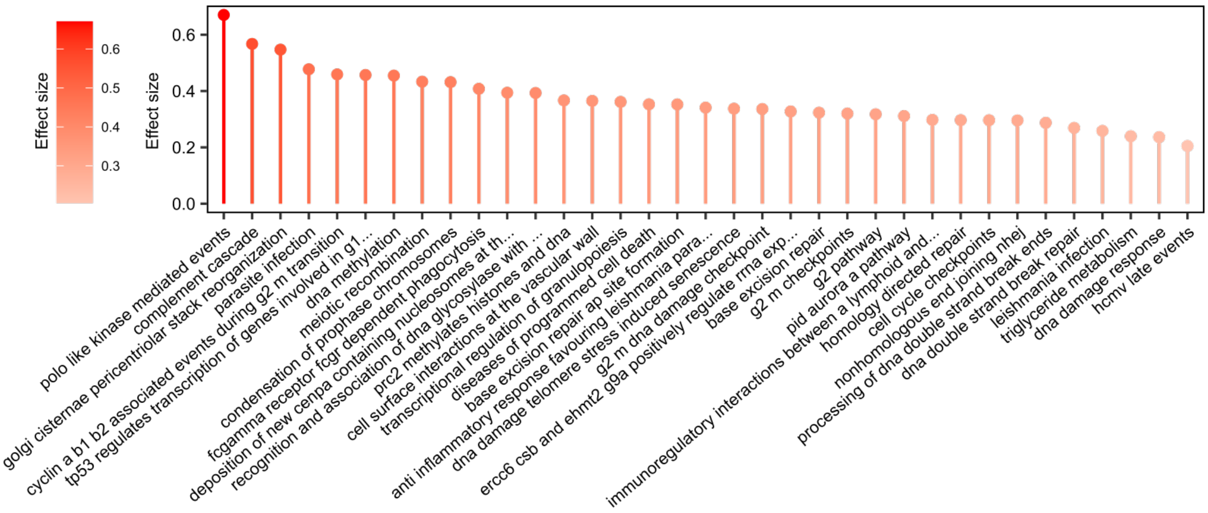

Supplementary Figure 5: Transcriptomic enrichment analysis comparing COVID-19 positive and negative samples. The 30 GSVA gene pathway enrichment terms with the greatest RRA scores (indicating consistent dysregulation in both the Wave 1 and Wave 2 transcriptomic datasets). All pathway terms shown were significantly enriched in the individual cohort analyses (1% FDR, LMM). Terms are ordered and coloured by their effect size. All terms were up-regulated in COVID-19 cases, so they are all coloured red.

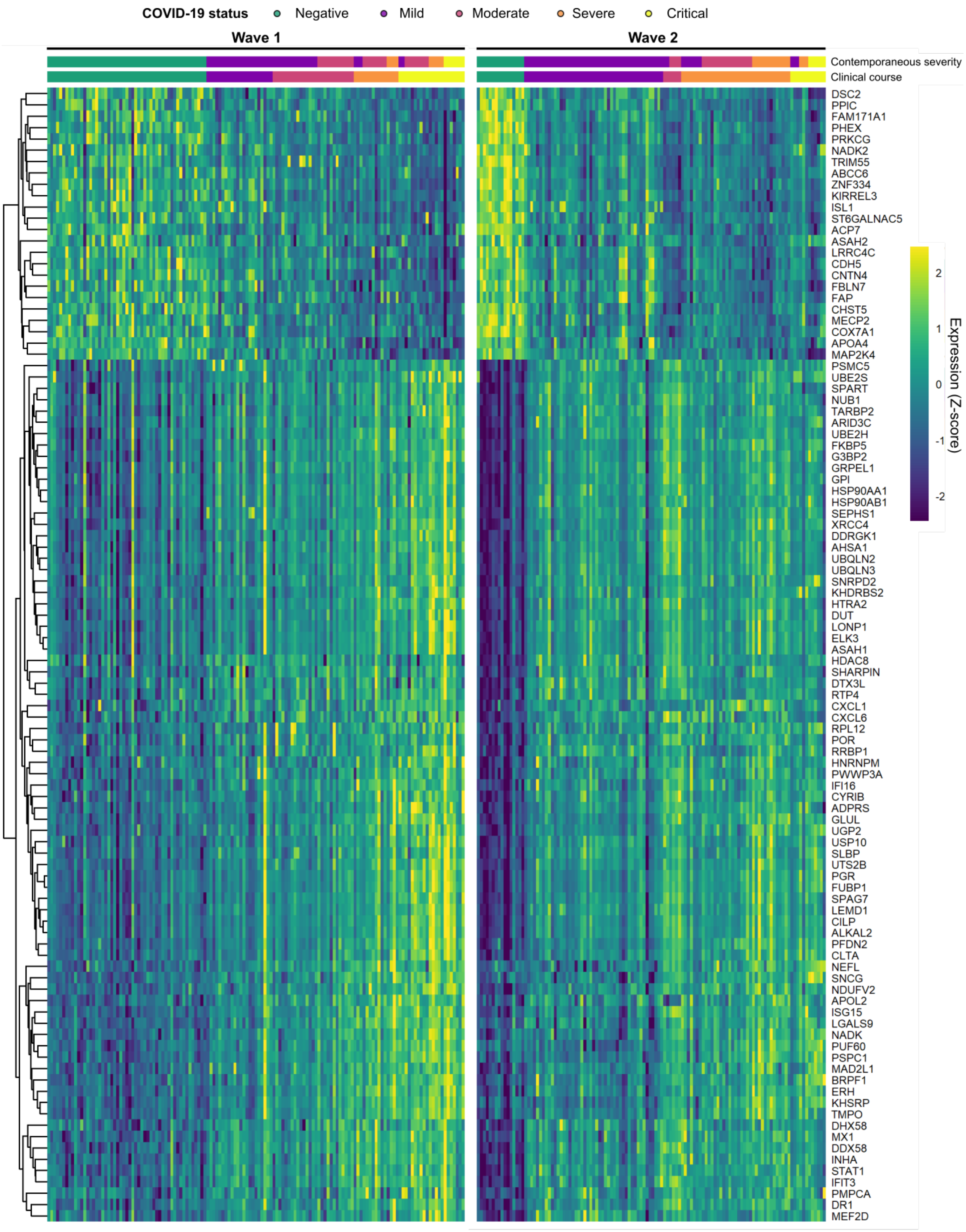

Supplementary Figure 6: Most significant differentially abundant plasma proteins in COVID-19 positive versus negative ESKD samples. Heatmap for the 100 proteomic features most significantly differentially abundant between COVID-19 cases and controls, according to robust rank aggregation (RRA). These proteins were significant in both cohorts (1% FDR, LMM). Columns are ordered by COVID-19 status and severity. For the Wave 1 heatmap, proteins are ordered by hierarchical clustering; the Wave 2 heatmap is ordered to match this. Each feature was scaled and centred separately in each dataset.

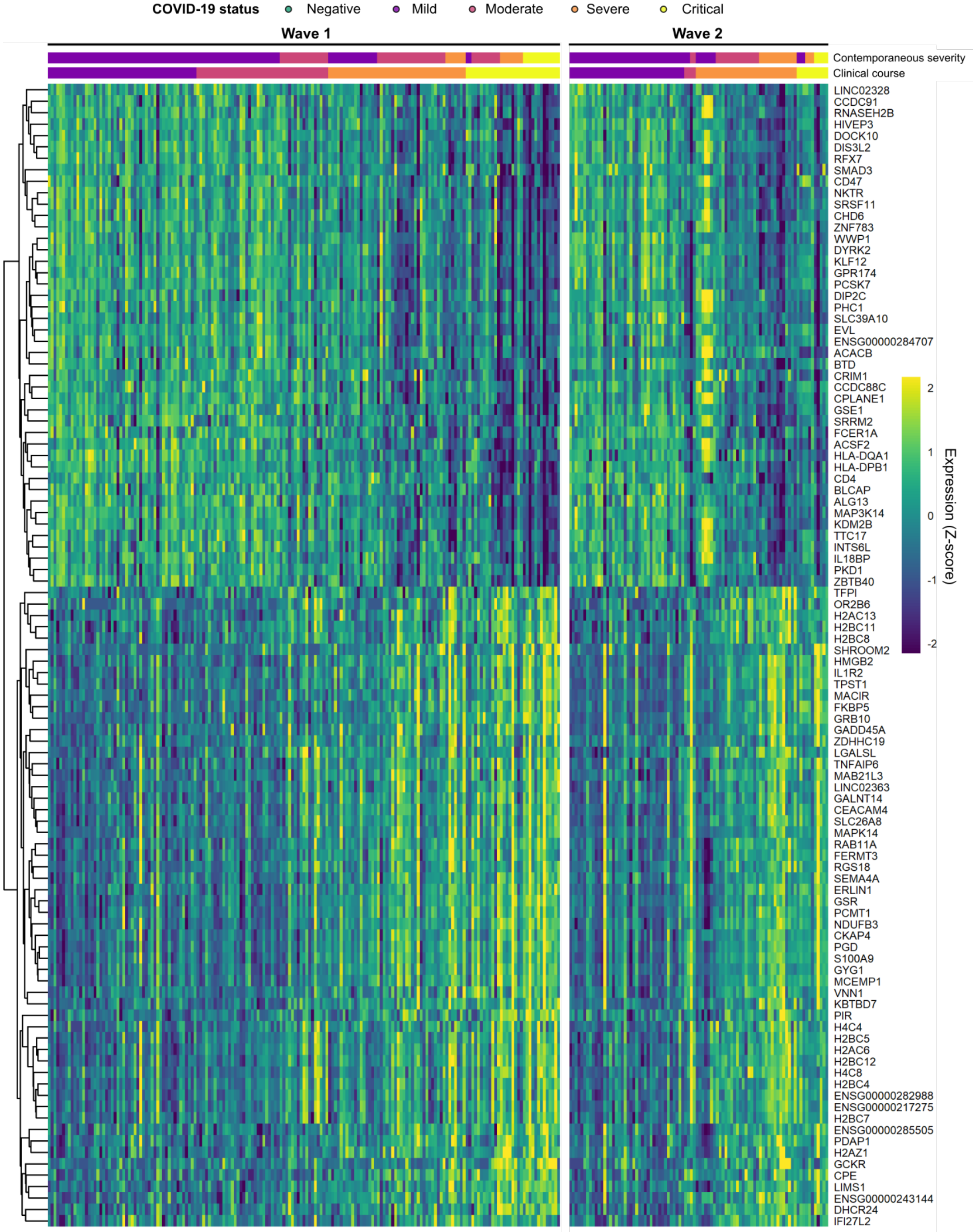

Supplementary Figure 7: Genes with the most significant association with COVID-19 severity. Heatmap for the 100 transcriptomic features most significantly associated with contemporaneous severity, according to robust rank aggregation (RRA). These genes were significant in both cohorts (1% FDR). Columns are ordered by COVID-19 status and severity. For the Wave 1 heatmap, genes are ordered by hierarchical clustering; the Wave 2 heatmap is ordered to match this. Each feature was scaled and centred separately in each dataset.

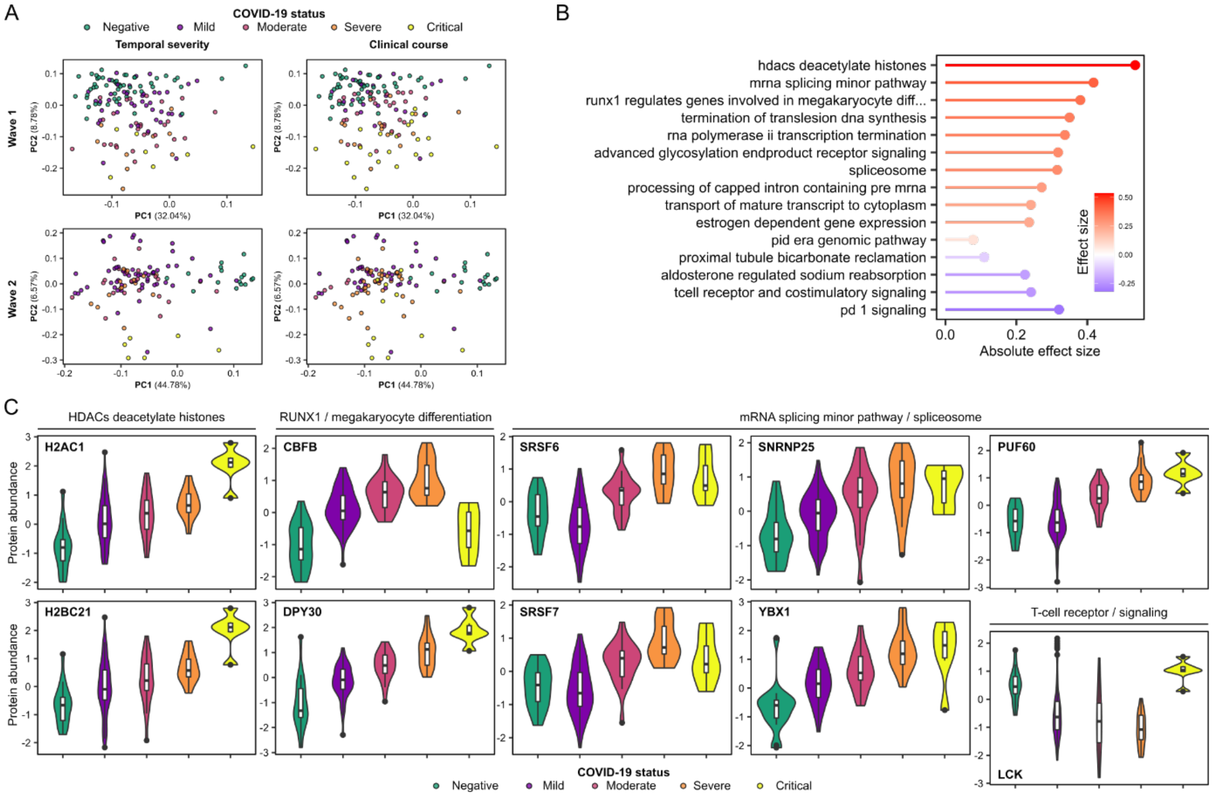

Supplementary Figure 8: Plasma proteomic associations with COVID-19 severity. A) PCA of the proteome. Each point represents a sample and is coloured by contemporaneous COVID-19 severity (left) and overall clinical course of the patient (right). **B**) The GSVA protein pathway enrichment terms significantly associated (1% FDR) with contemporaneous severity in both the Wave 1 and Wave 2 proteomic datasets. All pathway terms shown were significantly enriched in the individual cohort analyses (1% FDR). Terms are ordered and coloured by their effect size. Terms up-regulated in more severe COVID-19 are coloured red while down-regulated terms are blue. **C**) Violin plots show plasma protein abundance (Wave 2 cohort; 102 samples from 17 individuals and 16 pre-infection samples) stratified by COVID-19 status and severity (at time of sample) for selected proteins. All proteins shown were significantly associated (1% FDR) with contemporaneous severity in the Wave 2 cohort. Shaded areas indicate kernel density estimates. For boxplots, centre = median, upper bound = upper quartile (UQ), lower bound = lower quartile (LQ), upper whisker = largest value at most 1.5 * IQR (inter-quartile range) from the UQ, lower whisker = smallest value at most 1.5 * IQR from the LQ, points = samples outside of the range of the whiskers.

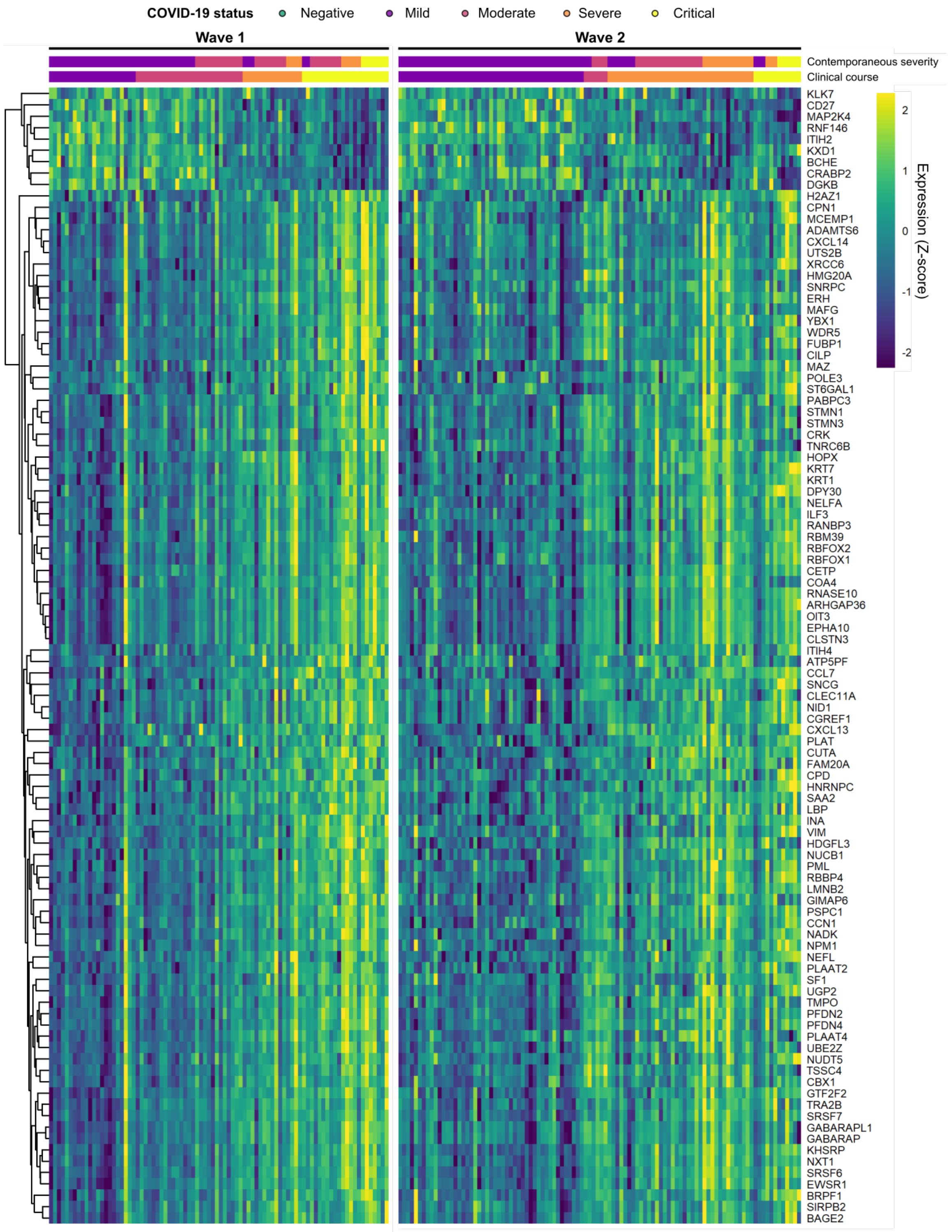

Supplementary Figure 9: Selected proteins associated with COVID-19 severity. Heatmap for the 100 proteomic features most significantly associated with contemporaneous WHO severity, according to robust rank aggregation (RRA). These proteins were significant in both cohorts (1% FDR). Columns are ordered by COVID-19 status and severity. For the Wave 1 heatmap, proteins are ordered by hierarchical clustering; the Wave 2 heatmap is ordered to match this. Each feature was scaled and centred separately in each dataset.

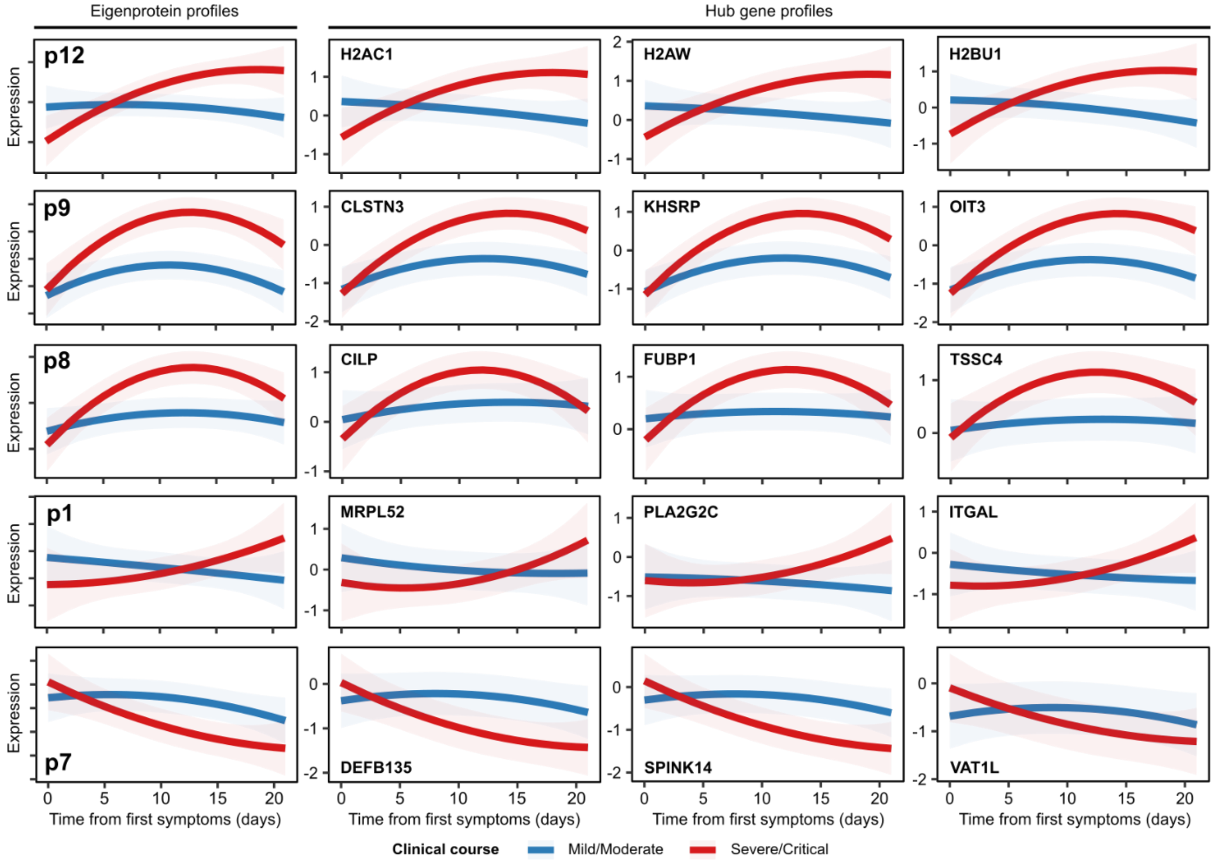

Supplementary Figure 10: Longitudinal profiles of protein modules and hub proteins. Modelled longitudinal profiles of protein modules with significant (5% FDR; LMM) TxCC interactions (left) and the profiles of their top three most central hub proteins (right). Lines represent LMM estimated marginal means and shaded areas represent their 95% confidence intervals. Red = patients with a severe/critical clinical course; blue = mild/moderate clinical course.

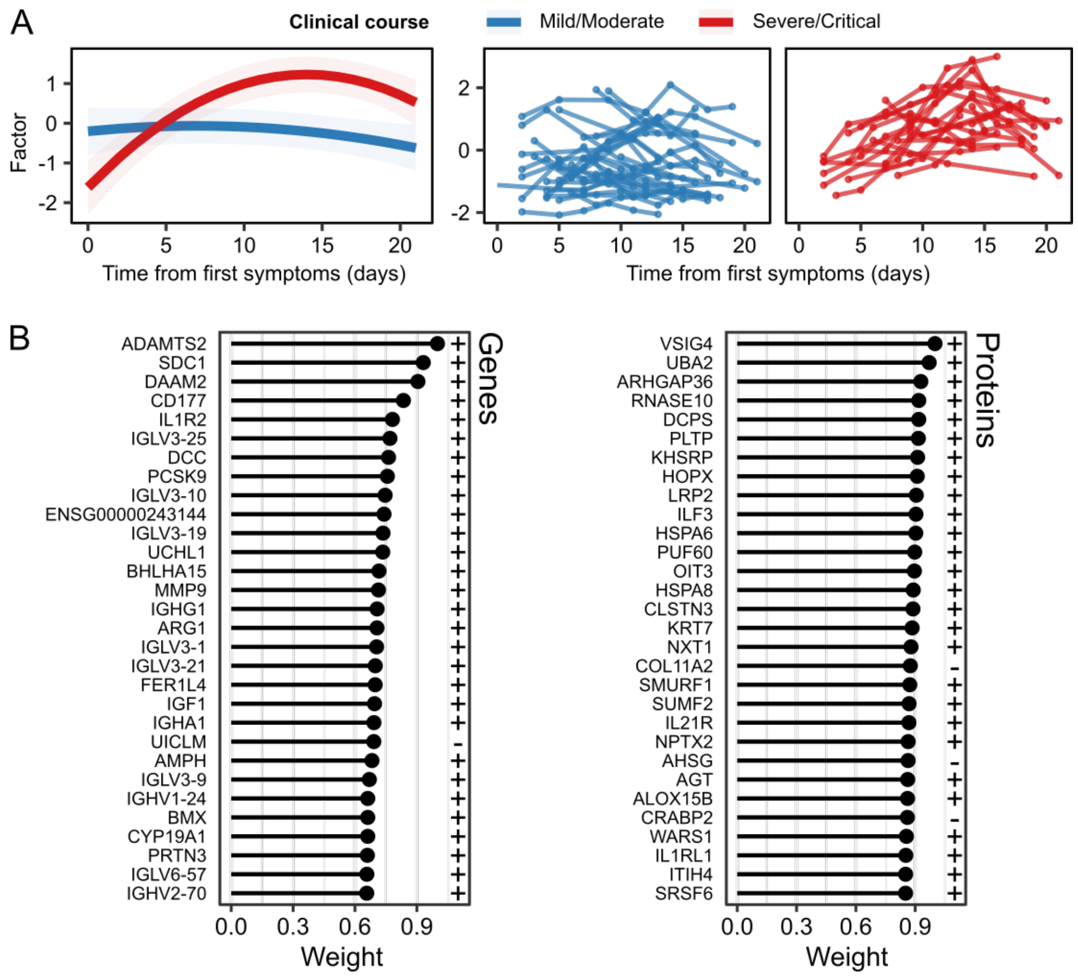

Supplementary Figure 11: A multi-modal factor representing COVID-19 severity. A) The longitudinal profile of a factor identified by MEFISTO with a significant (5% FDR; LMM) TxCC interaction. Lines represent estimated marginal means and shaded areas represent their 95% confidence intervals (left) and the trajectory of the factor for each individual (right). **B**) The weights of individual genes and proteins with respect to the factor identified by MEFISTO. A plus sign indicates molecules positively associated with the factor while molecules negatively associated with the factor have a minus sign.

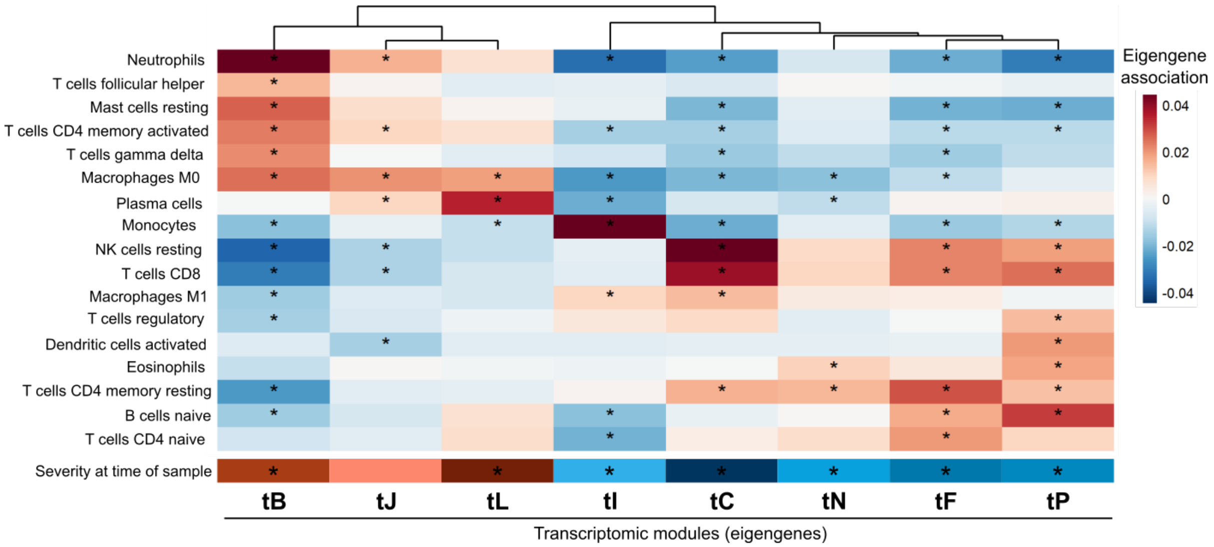

Supplementary Figure 12: Association of transcriptomic modules with imputed cell abundances. LMM association of modules (columns) with cell abundances (rows) imputed using CIBERSORTx. All modules displayed have significant TxCC interaction effects (5% FDR, LMM). The bottom row shows the LMM association with severity score at time of sample. Asterisks represent significant (5% FDR) associations.

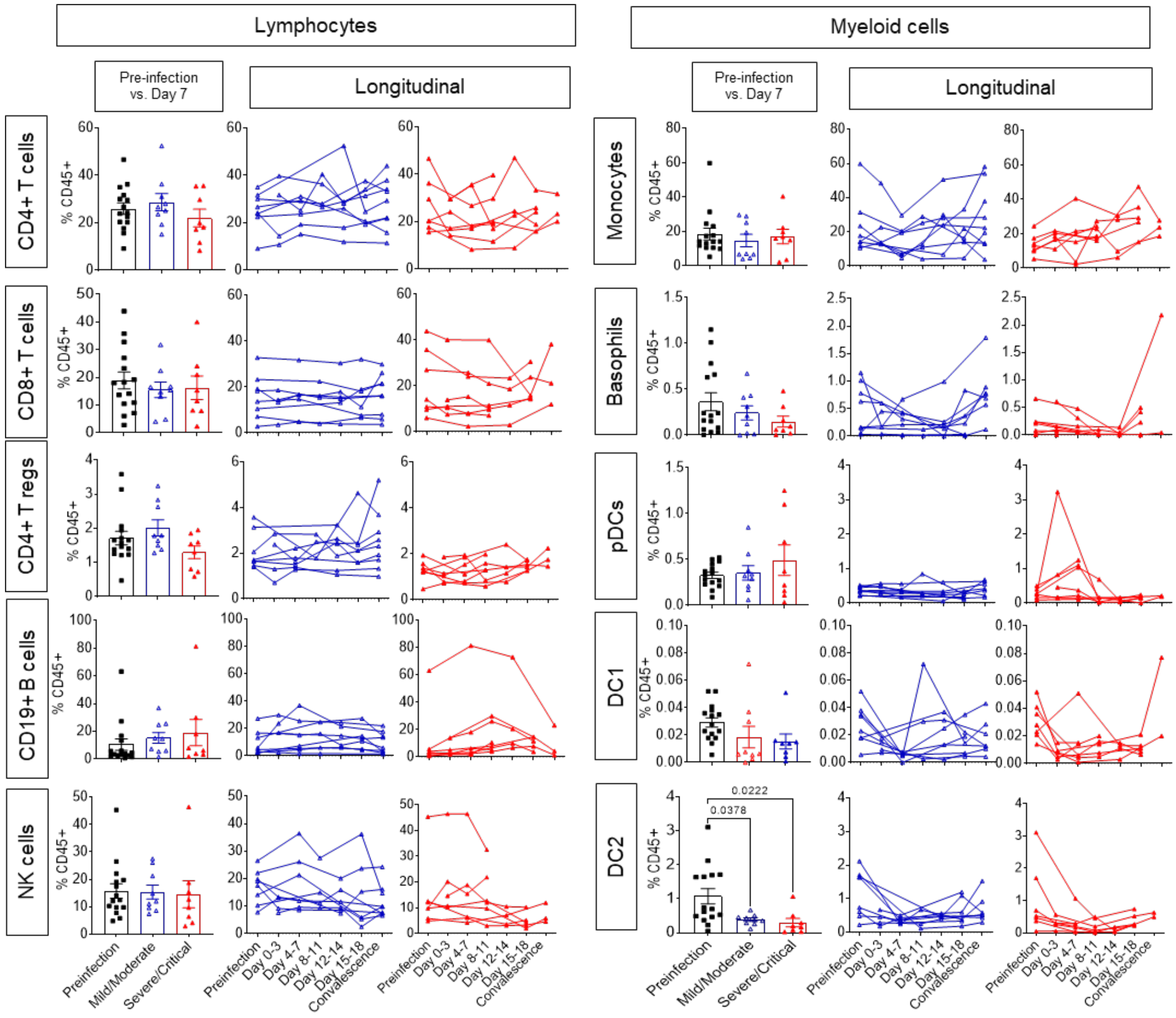

Supplementary Figure 13: Proportion of major lymphocytes and myeloid cells in PBMCs (Wave 2 cohort).

Panels marked Pre-infection vs. Day 7: frequencies of major immune subsets gated on CD45+ leukocytes in samples taken approximately 8-9 months prior to infection (Pre-infection n=15 patients, black squares, each symbol represents an individual) and samples from the same individuals at closest sample to day 7 from COVID-19 symptom onset, stratified by disease course: mild/moderate (n=9 patients, blue triangles) or severe/critical course (n=8 patients, red triangles). Data presented as mean (box) ± S.E.M (whiskers).

Panels marked Longitudinal: frequencies of major immune subsets before, during and after infection as a line plot. Each line depicts one individual. Blue = patients with a mild/moderate clinical course; sample numbers: Pre-infection n=8, Day 0-3 n=4, Day 4-7 n=6, Day 8-11 n=4, Day 12-14 n=6, Day 15-18 n=5, Convalescence n=9 (sample numbers vary due to insufficient cell counts). Red= severe/critical clinical course; sample numbers: Pre-infection n=7, Day 0-3 n=5, Day 4-7 n=6, Day 8-11 n=6, Day 12-14 n=4, Day 15-18 n=5, Convalescence n=3. One-way ANOVA with Dunnet's correction for multiple comparisons was used for statistical analysis. Only significant differences are indicated. *p <0.05. NK= natural killer; pDCs= plasmacytoid dendritic cells. DC= dendritic cells.

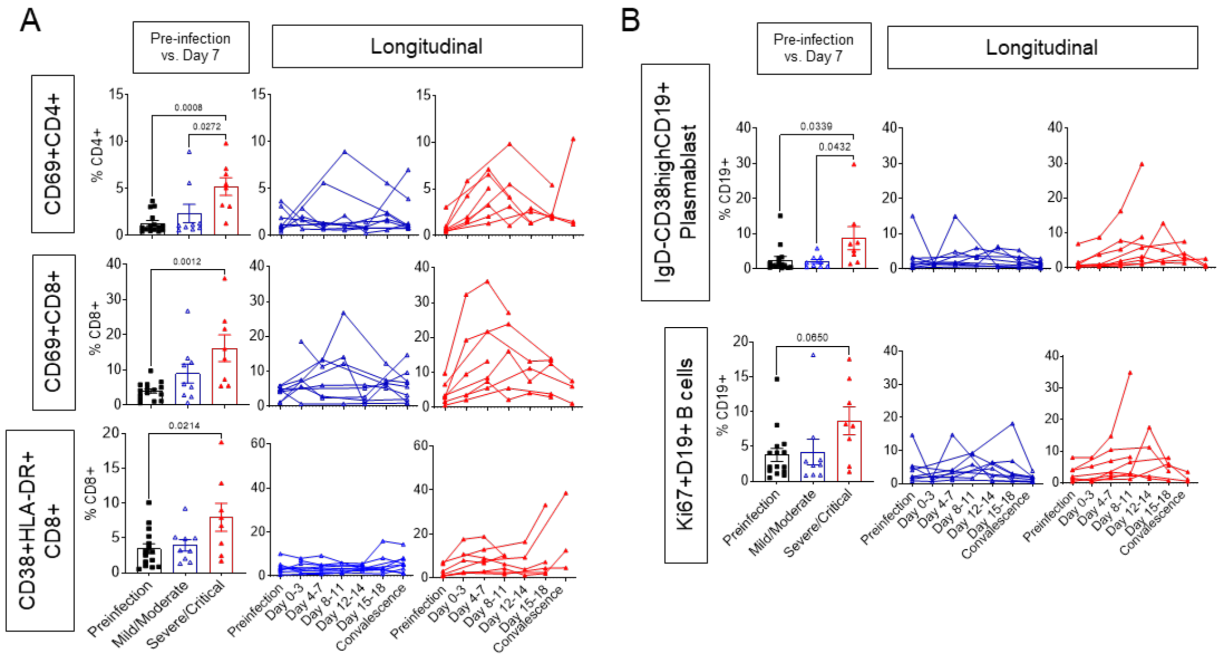

Supplementary Figure 14: Increased activated T cells, plasmablast and proliferating B cells is associated with disease severity in COVID-19 ESKD patients.

A) Percentage of cells expressing activation markers CD69, CD38 and HLA-DR gated on CD4+ T cells or CD8+ T cells. Panel marked Pre-infection vs. Day 7: data plotted for samples taken prior to infection (Pre-infection n=15) and for closest sample to day 7 from symptom onset (Mild/Moderate n=9; Severe/Critical n=8).

B) Frequencies of IgD-CD38^{hi}CD19+ plasmablast and Ki67+ cells gated on CD19+ B cells. Panel marked Pre-infection vs. Day 14: data plotted for samples taken prior to infection (Pre-infection n=15) and for closest sample to day 14 from symptom onset (Mild/Moderate n=9; Severe/Critical n=8).

(**A-B**) Left panels: data presented as mean (bar) ± S.E.M (whiskers). Each symbol represents an individual. Right panels: frequencies of cells over the course of infection as a line plot. Each continuous line depicts one individual. Mild/moderate patients in blue (Pre-infection n=8, Day 0-3 n=4, Day 4-7 n=6, Day 8-11 n=4, Day 12-14 n=6, Day 15-18 n=5, Convalescence n=9) and severe/critical in red (Pre-infection n=7, Day 0-3 n=5, Day 4-7 n=6, Day 8-11 n=6, Day 12-14 n=4, Day 15-18 n=5, Convalescence n=3) (sample numbers vary due to insufficient cell counts). One-way ANOVA with Dunnet's correction for multiple comparisons used for statistical analysis. Only significant differences are indicated ($p < 0.05$).

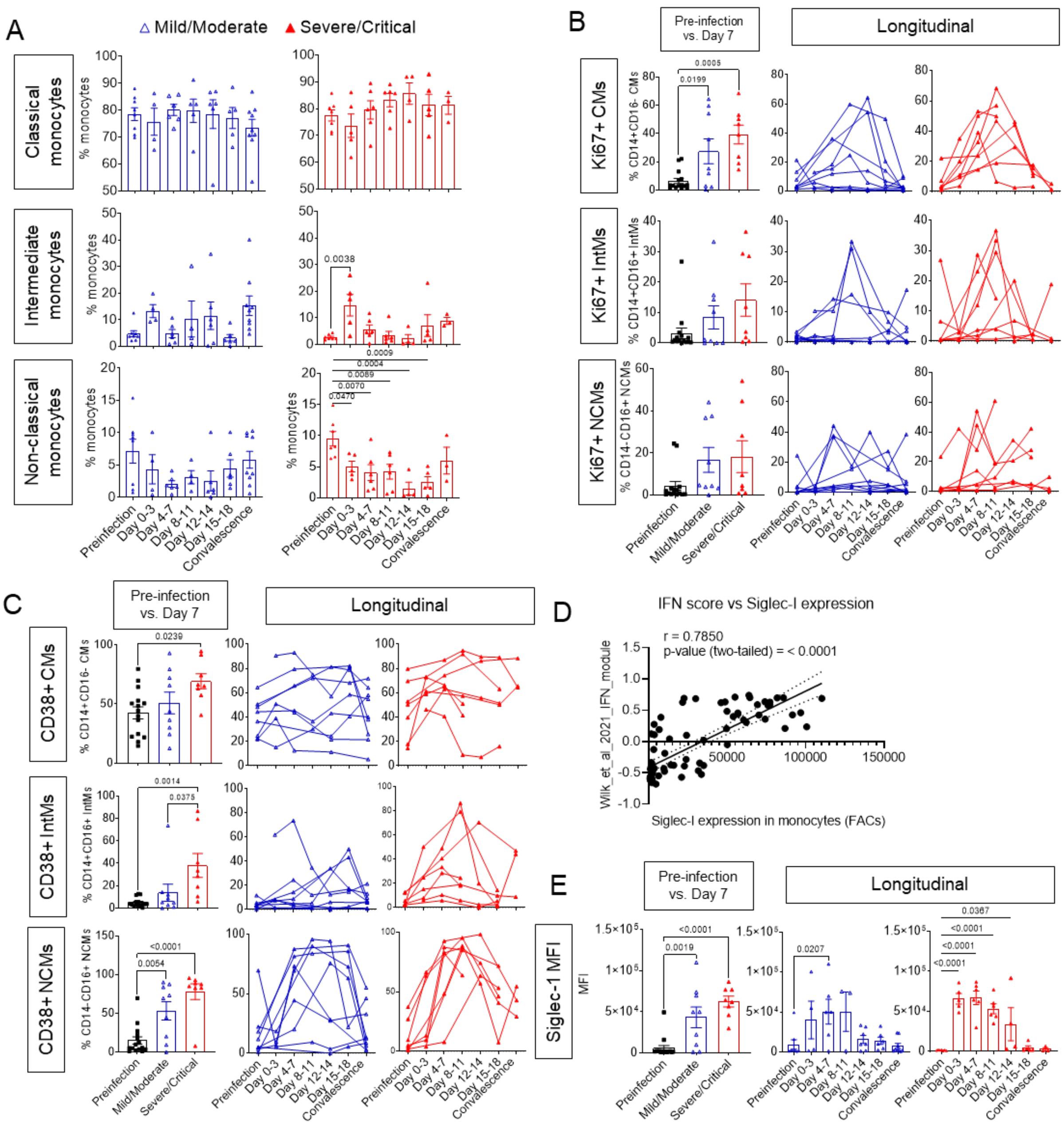

Supplementary Figure 15: Increased activated and proliferating circulatory monocytes is associated with disease severity in COVID-19 ESKD patients.

A) Frequencies of monocyte subsets as defined with CD14+ and CD16+ expression gated on total monocytes. Percentage of cells expressing CD38 (**B**) and Ki67 (**C**) gated on CD14+CD16- CMs, CD14+CD16+ IntMs, CD14-CD16+ NCMs. **D)** Correlation (Pearson's r) of SIGLEC-1 protein expression by MFI to RNAseq-derived GSVA enrichment score for type I IFN signatures. **E)** Siglec-1 expression in MFI gated on total monocytes.

(**B, C, E**): Data plotted for samples taken prior to infection (Pre-infection n=15) and for closest sample to day 7 from symptom onset (Mild/Moderate n=9; Severe/Critical n=8). Data presented as mean ± S.E.M. Each symbol represents an individual.

(**A, B, C, E**) Frequencies of cells over the course of infection as a line or bar plot. Mild/moderate patients in blue (Pre-infection n=8, Day 0-3 n=4, Day 4-7 n=6, Day 8-11 n=4, Day 12-14 n=6, Day 15-18 n=5, Convalescence n=9) and severe/critical in red (Pre-infection n=7, Day 0-3 n=5, Day 4-7 n=6, Day 8-11 n=6 Day 12-14 n=4, Day 15-18 n=5, Convalescence n=3) (sample numbers vary due to insufficient cell counts). One-way ANOVA with Dunnet's correction for multiple comparisons used for statistical analysis. Only significant differences are indicated ($p < 0.05$).
CM=Classical Monocytes; IntM=Intermediate Monocytes; NCM=Non-classical Monocytes; MFI=Median Fluorescence Intensity.

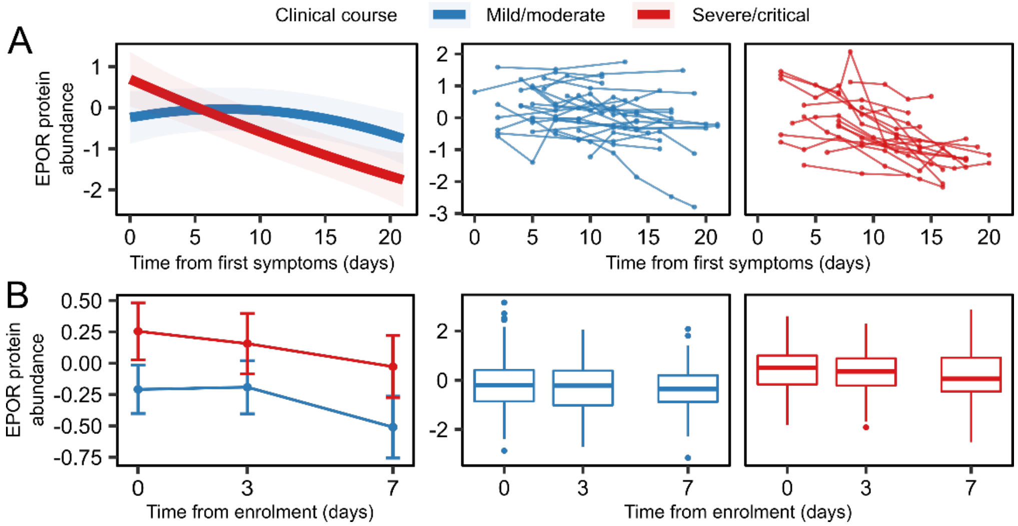

Supplementary Figure 16. The longitudinal profile of plasma EPOR protein. A) The temporal profile of EPOR in our study of ESKD patients, stratified by severity of the patients' overall clinical course. Left: lines represent LMM estimated marginal means and shaded areas represent their 95% confidence intervals. Right: raw data for each individual (n=169 samples from 40 individuals). **B**) The profile of EPOR in the data from Filbin *et al.* (non-ESKD cohort; n=655 samples from 306 individuals). Left: points represent LMM estimated marginal means and error bars indicate their 95% confidence intervals. Right: box and whiskers plots showing the underlying protein data, where centre = median, upper bound = upper quartile (UQ), lower bound = lower quartile (LQ), upper whisker = largest value at most 1.5 * IQR (inter-quartile range) from the UQ, lower whisker = smallest value at most 1.5 * IQR from the LQ, points = samples outside the range of the whiskers.

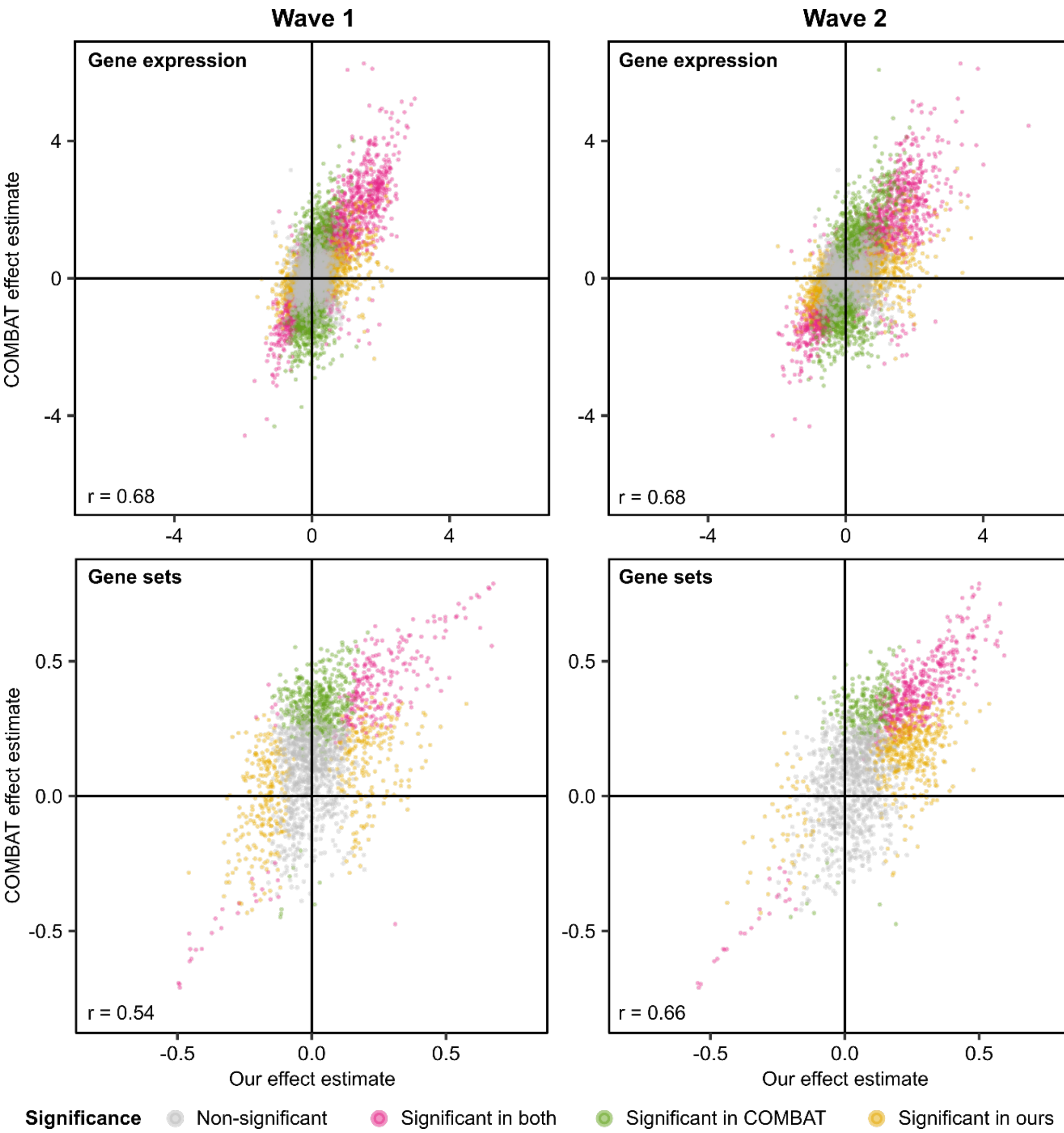

Supplementary Figure 17. Comparison to the data published by the COMBAT Consortium. The effect estimates for the differential expression of genes between COVID-19 positive vs. negative samples were compared between the cohorts in this study (ESKD patients) and a re-analysis of the data published by the COvid-19 Multi-omics Blood ATlas (COMBAT) Consortium. Colour represents statistical significance (FDR <0.01) in each study. The Pearson's correlation (r) is displayed for each comparison.

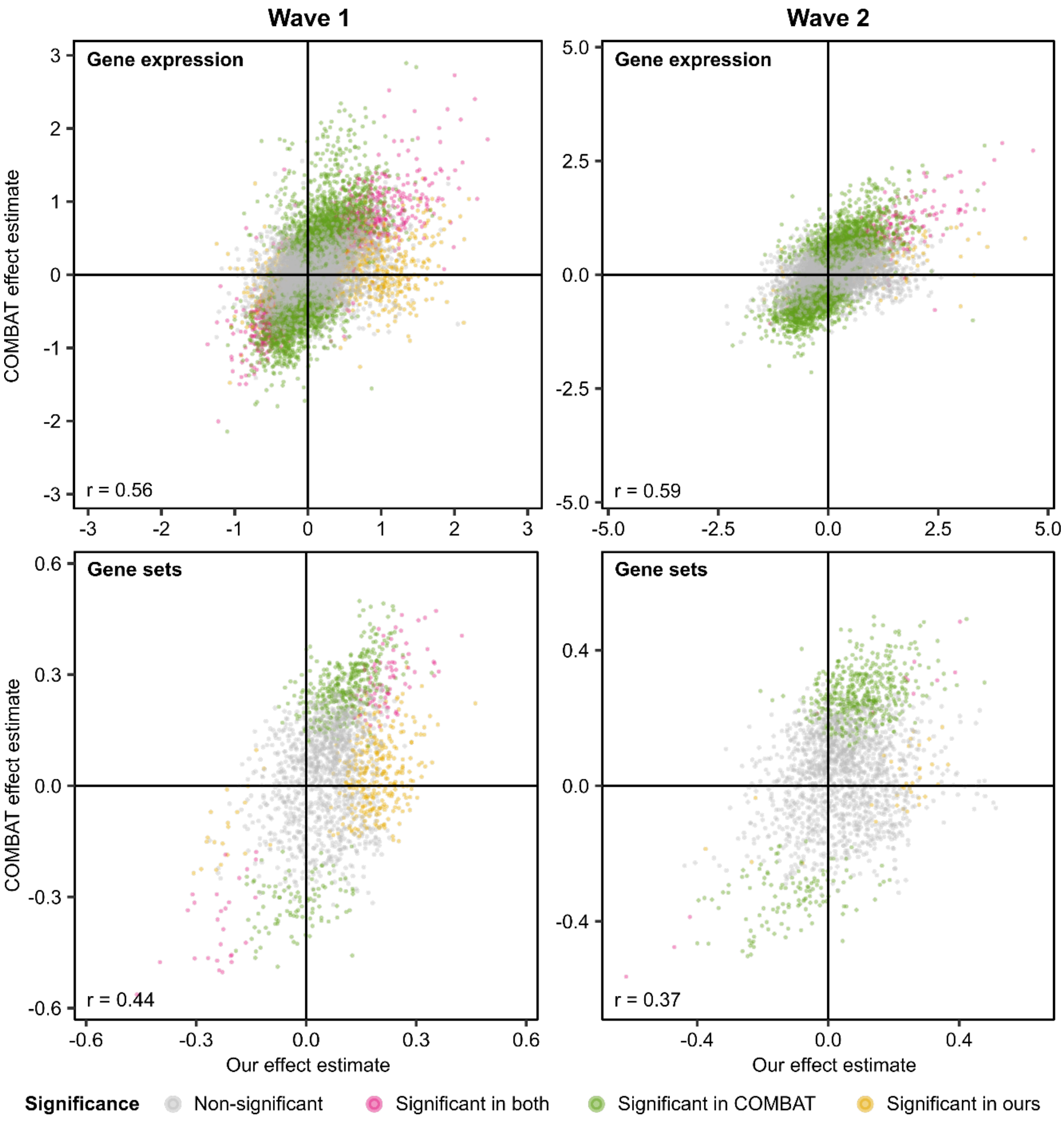

Supplementary Figure 18. Comparison to the data published by the COMBAT Consortium. The effect estimates for the association of genes contemporaneous COVID-19 severity were compared between the cohorts in this study and a re-analysis of the data published by the COvid-19 Multi-omics Blood ATlas (COMBAT) Consortium. Colour represents significance (FDR <0.01) in each study. The Pearson's correlation (r) is displayed for each comparison.

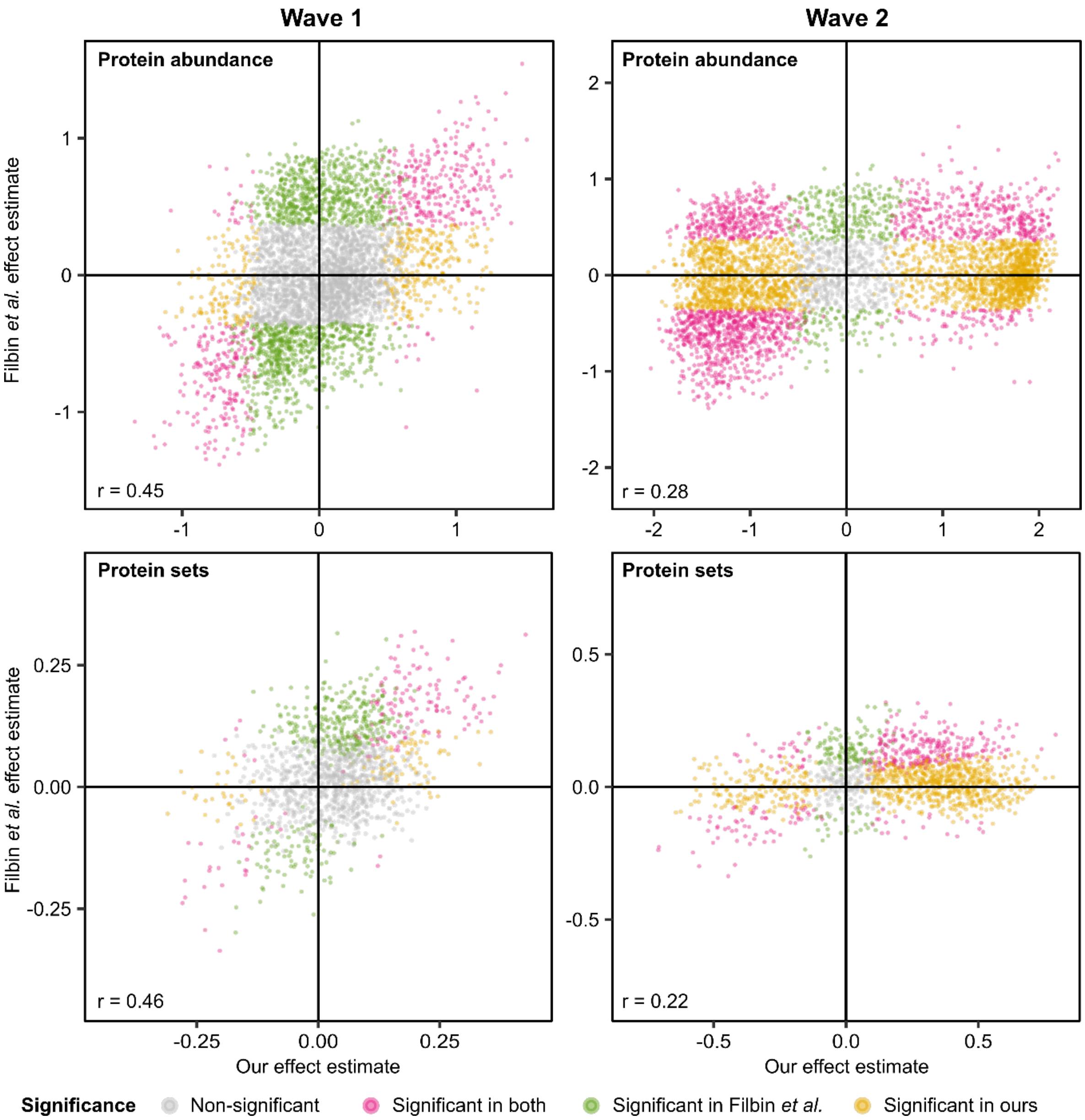

Supplementary Figure 19. Comparison to the data published by Filbin *et al.* The effect estimates for the differential abundance of proteins between COVID-19 positive vs. negative patients were compared between the cohorts in this study and a re-analysis of the data published by Filbin *et al*. Colour represents significance (FDR <0.01) in each study. The Pearson's correlation (r) is displayed for each comparison.

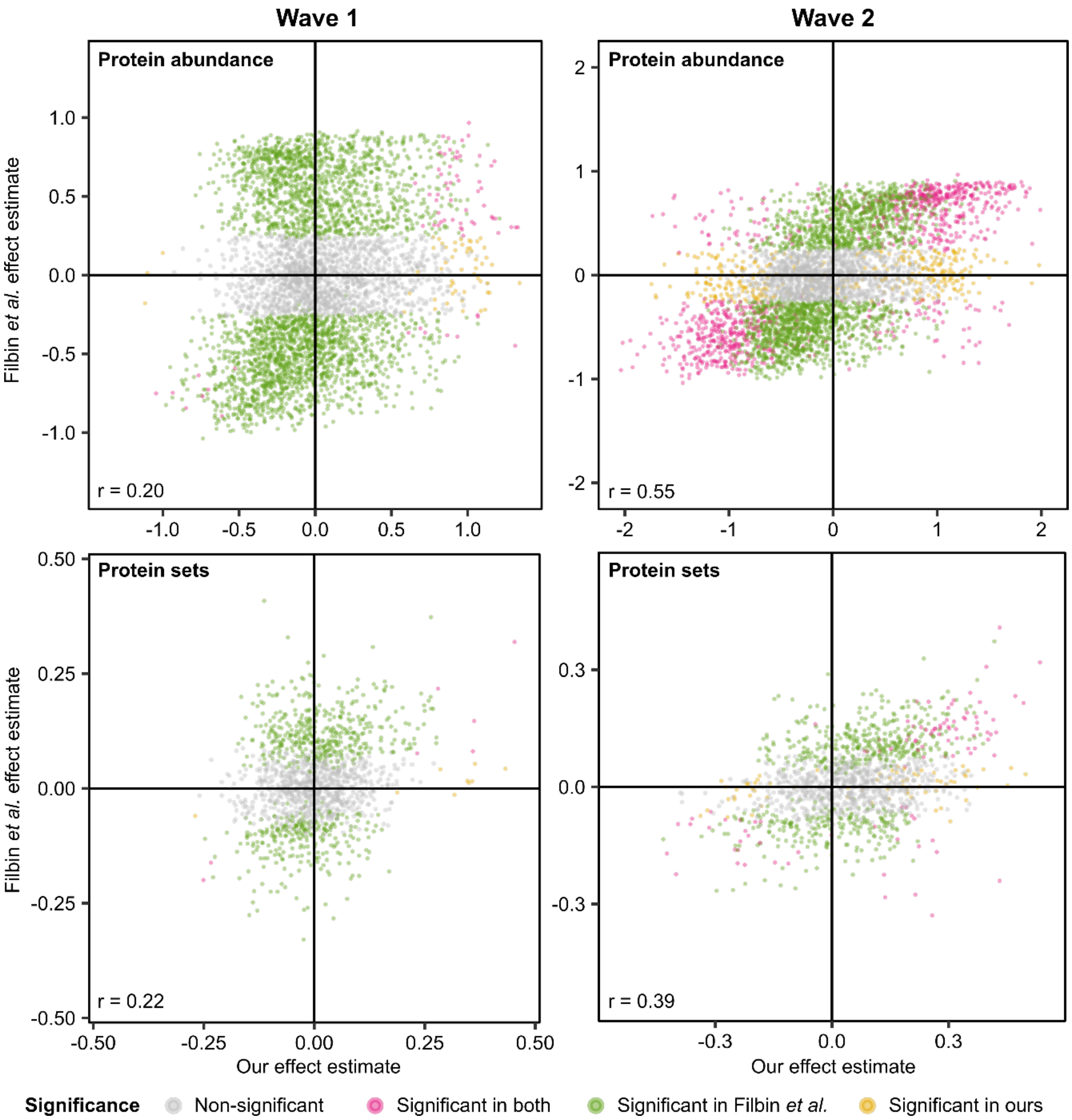

Supplementary Figure 20. Comparison to the data published by Filbin *et al.* The effect estimates for the association of proteins with COVID-19 severity were compared between the cohorts in this study and a re-analysis of the data published by Filbin *et al*. Colour represents significance (FDR <0.01) in each study. The Pearson's correlation (r) is displayed for each comparison.

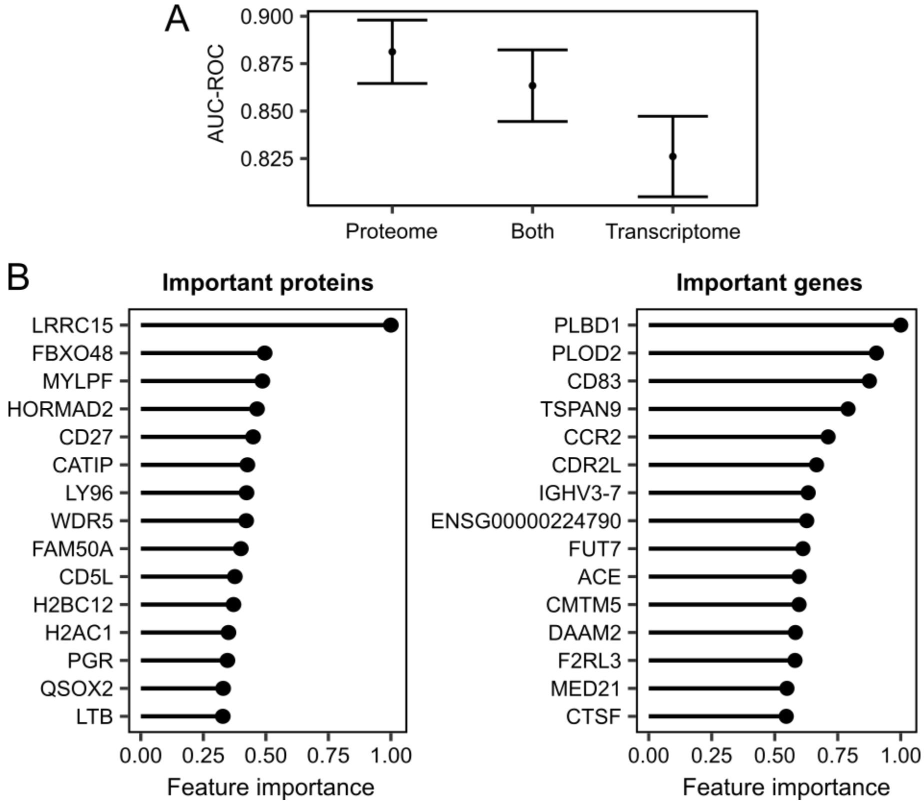

Supplementary Figure 21: Predicting COVID-19 severity using random forests. **A**) Point estimates (mean) and 95% confidence intervals of area under the curve from receiver operating characteristic curves (AUC-ROC) for predicting COVID-19 severity (from 51 independent samples i.e. one sample per patient) using random forests. Both = supervised learning on the combined proteomic and transcriptomic data. **B**) Important proteins (left) and genes (right) for the random forests model. Feature importance is scaled between 0 and 1, where 1 represents the most important feature.

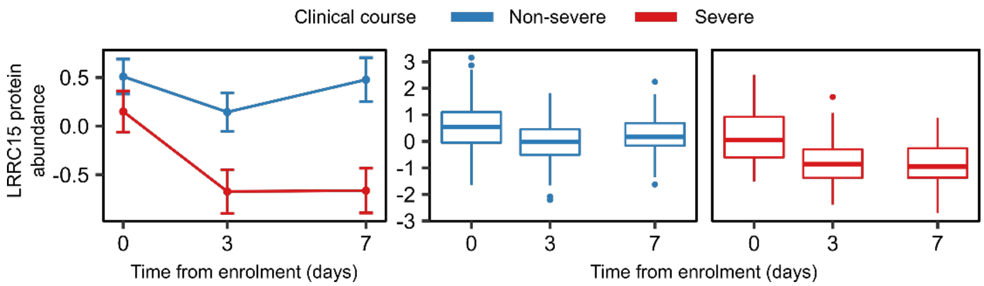

Supplementary Figure 22. The longitudinal profile of LRRC15 in the data published by Filbin *et al*. The profile of LRRC15 plasma protein concentration over time, stratified by severity of the patients' overall clinical course (n=655 samples from 306 individuals). Left: points represent LMM estimated marginal means and error bars indicate their 95% confidence intervals. Right: box and whiskers plots showing the underlying protein data, where centre = median, upper bound = upper quartile (UQ), lower bound = lower quartile (LQ), upper whisker = largest value at most 1.5 * IQR (inter-quartile range) from the UQ, lower whisker = smallest value at most 1.5 * IQR from the LQ, points = samples outside of the range of the whiskers.

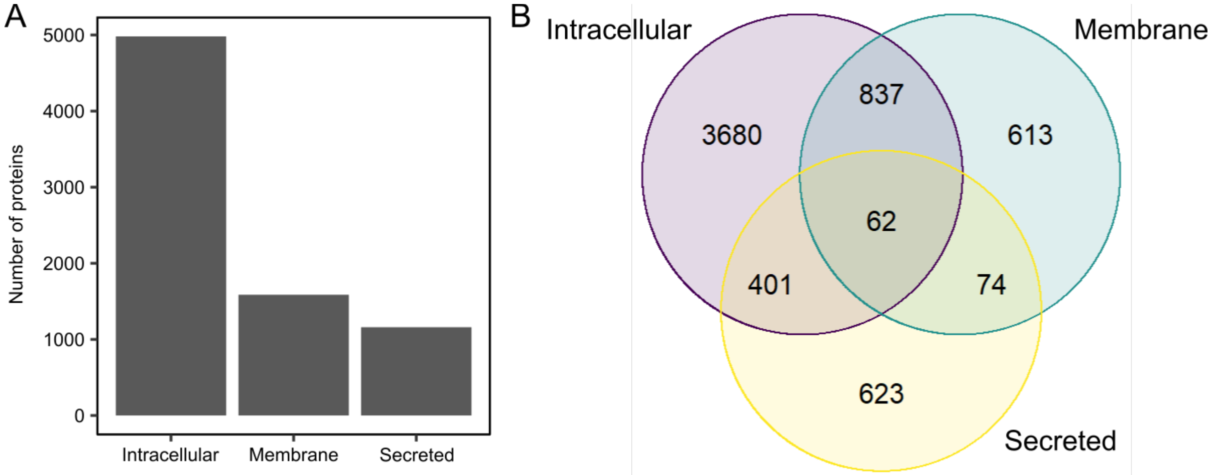

Supplementary Figure 23: Human Protein Atlas classification of proteins measured by the SomaScan v4.1 assay. A) The number of unique proteins measured that were labelled as intracellular, membrane and secreted. **B**) Venn diagram illustrating overlap between the annotations.

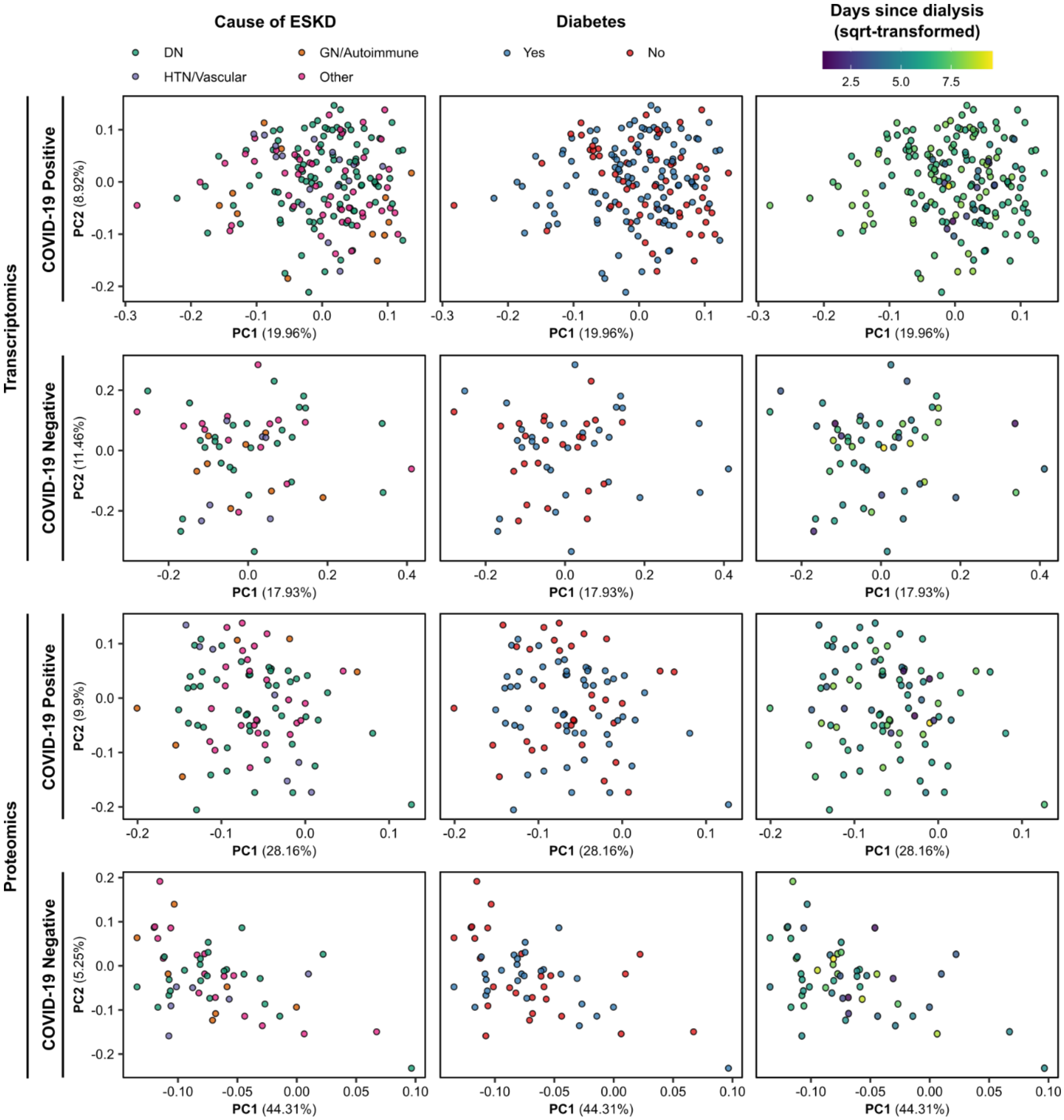

Supplementary Figure 24: Principal components analysis (PCA) to visualise the impact of clinical covariates on PBMC transcriptome and plasma proteome. PCA plots of the transcriptome and proteome in the Wave 1 cohort, stratified by COVID-19 positive and negative samples. Samples are coloured according to clinical covariates. Left panel: cause of ESKD (DN = diabetic nephropathy, GN = glomerulonephritis, HTN = hypertension). Middle panel: diabetes status (all bar one of the diabetic patients had type 2 diabetes). Right panel: time since first initiation of haemodialysis (measured as the square root of days since the patient first began haemodialysis). The variance explained by PC1 and 2 is indicated in parentheses.

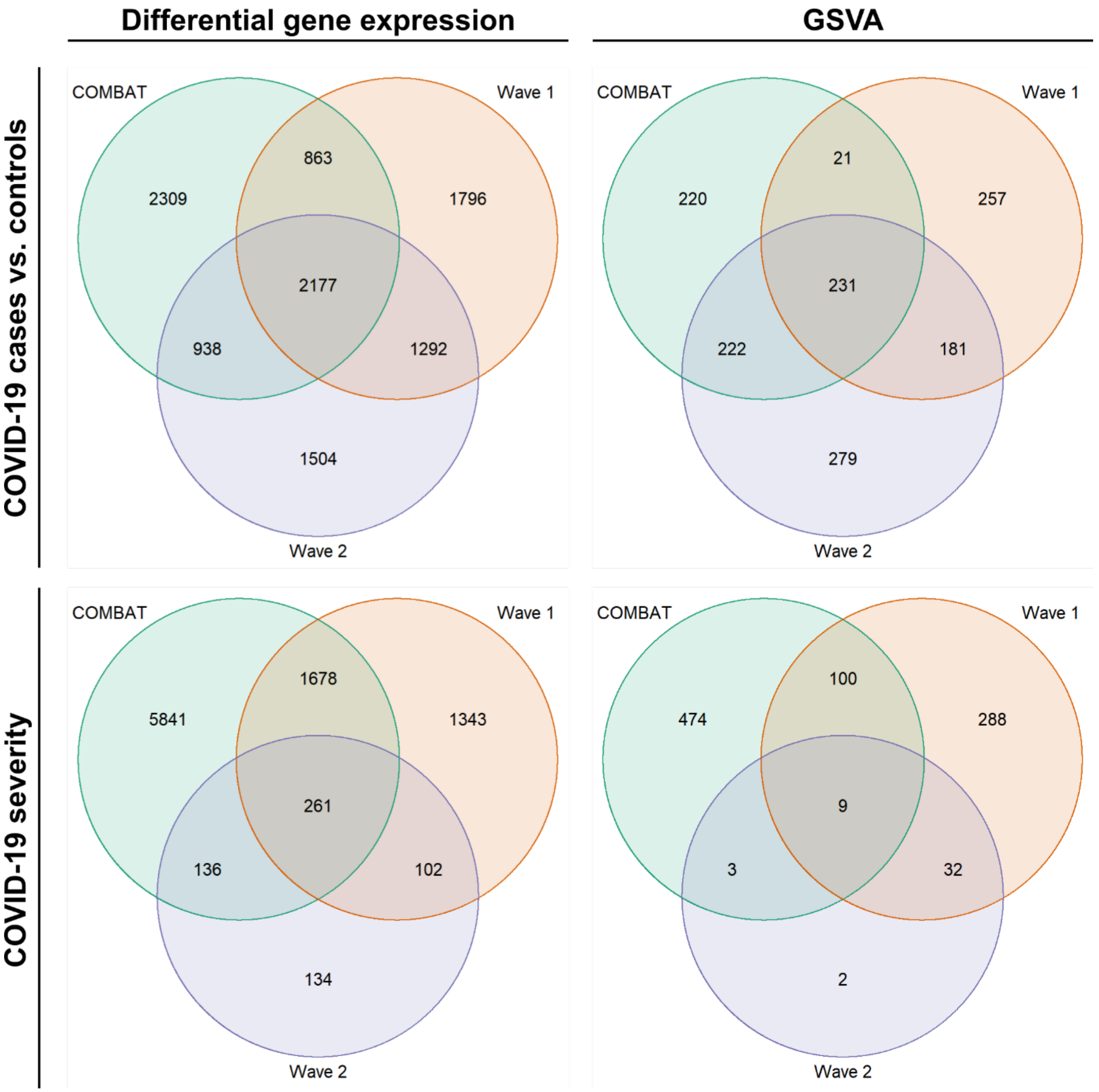

Supplementary Figure 25. Overlap of significant genes and gene sets with data from the COMBAT study. Genes and GSVA gene sets with Benjamini-Hochberg adjusted P-values of less than 0.01 were considered significant in each cohort. The upper panels compare COVID-19 positive versus negative samples. The lower panels compare associations with COVID-19 severity. Statistical testing was performed using LMMs.

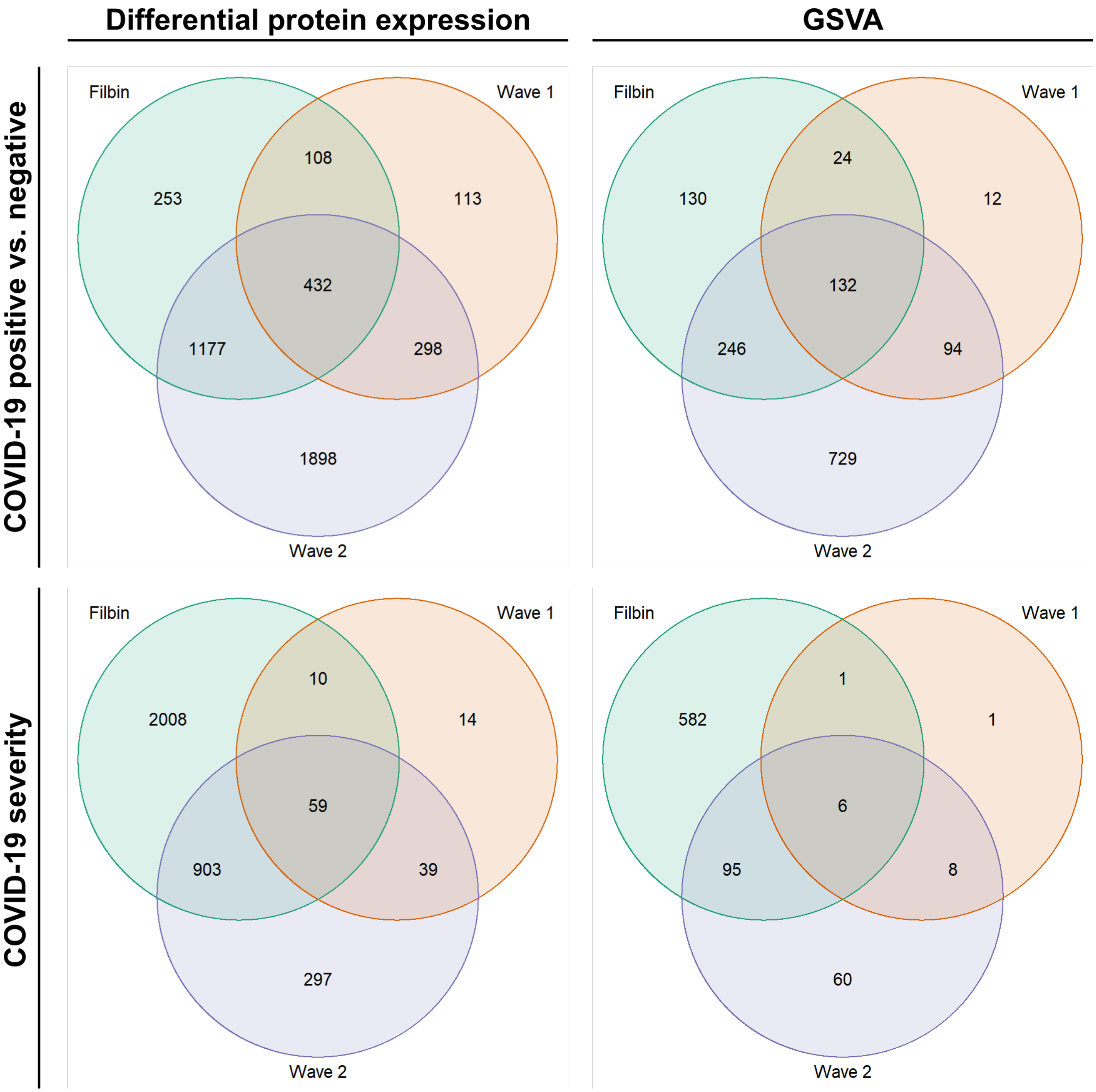

Supplementary Figure 26. Overlap of significant proteins and protein sets with data of Filbin *et al*. Proteins and GSVA protein sets with Benjamini-Hochberg adjusted P-values of less than 0.01 were considered significant in each cohort. The upper panels compare COVID-19 positive versus negative samples. The lower panels compare associations with COVID-19 severity. Statistical testing was performed using LMMs.

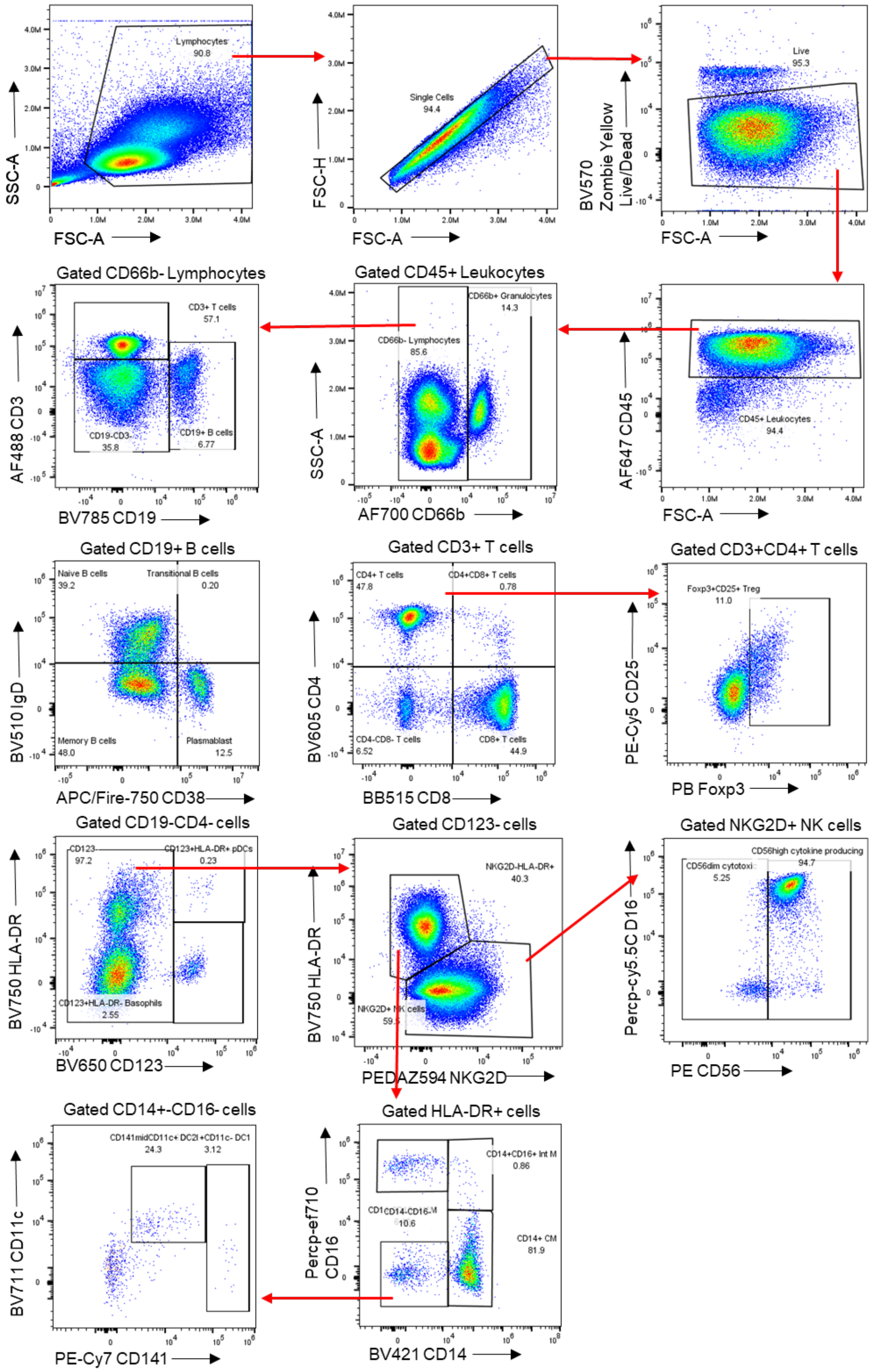

Supplementary Figure 27: Gating strategy for flow cytometry analysis of myeloid and lymphoid cell subsets. Percentages of immune cells from cryopreserved PBMC samples were characterized by flow cytometry. Representative data from one sample are shown. Gates were positioned according to isotype controls. Red arrows indicate the order gating was applied on samples.

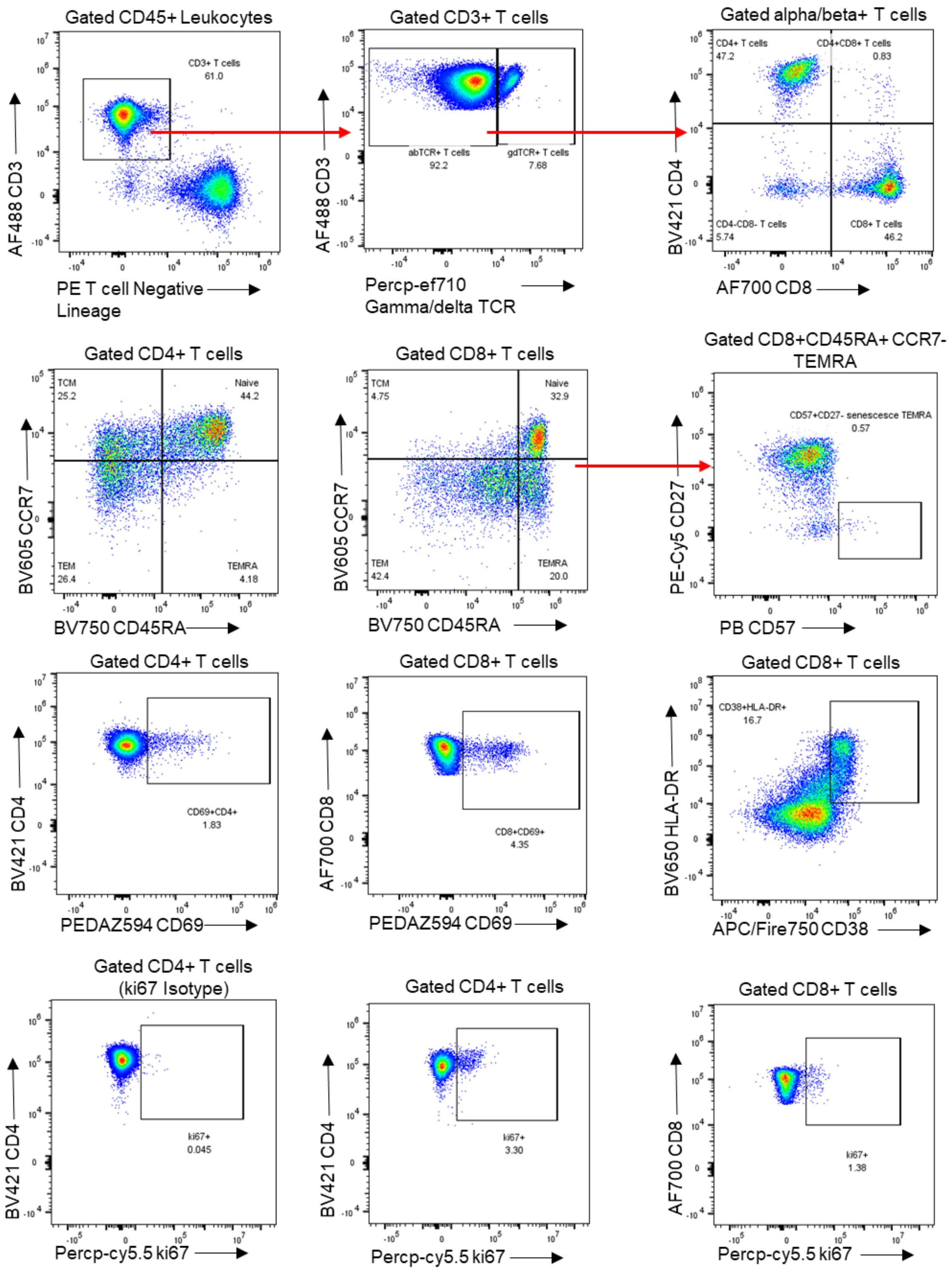

Supplementary Figure 28: Gating strategy for flow cytometry analysis of additional T cell subsets. Percentages of immune cells from cryopreserved PBMC samples were characterized by flow cytometry. Representative data from one sample are shown. Gates were positioned according to isotype controls. Red arrows indicate the order gating was applied on samples

Supplementary Tables

Supplementary Table 1: Characteristics of the Wave 1 cohort.

	Overall (n = 53)	Peak severity mild or moderate (n = 28)	Peak severity severe or critical (n = 25)
**Age**			
**Median**	72.0	73.0	68.0
**(IQR)**	62.0-76.0	64.8-76.2	62.0-76.0
**Sex**			
**M**	37 (69.8%)	18 (64.3%)	19 (76.0%)
**F**	16 (30.2%)	10 (35.7%)	6 (24.0%)
**Ethnicity**			
**Asian**	21 (39.6%)	11 (39.3%)	10 (40.0%)
**White**	17 (32.1%)	6 (21.4%)	11 (44.0%)
**Black**	8 (15.1%)	5 (17.9%)	3 (12.0%)
**Other**	7 (13.2%)	6 (21.4%)	1 (4.0%)
**Diabetes**	32* (60.4%)	16* (57.1%)	16 (64.0%)
**Current smoker**	1 (1.9%)	1 (3.6%)	0 (0.0%)
**ESKD cause**			
**DN**	27 (50.9%)	14 (50.0%)	13 (52.0%)
**HTN/vascular**	5 (9.4%)	3 (10.7%)	2 (8.0%)
**GN/autoimmune**	3 (5.7%)	1 (3.6%)	2 (8.0%)
**Genetic**	1 (1.9%)	1 (3.6%)	0 (0.0%)
**Other/unknown**	17 (32.1%)	9 (32.1%)	9 (36.0%)
**Hospitalisation due to COVID-19†**	32 (60.4%)	7 (25.0%)	25 (100%)
**Fatal COVID-19**	9 (17.0%)	0 (0.0%)	9 (36.0%)

DN = diabetic nephropathy. GN = glomerulonephritis. HTN = hypertension. IQR = inter-quartile range. Subsets defined according to peak WHO severity over the course of the illness.

*One patient had type 1 diabetes, the remainder type 2. †3 patients were hospitalised prior to COVID-19 diagnosis. 8 patients diagnosed with COVID-19 as outpatients subsequently deteriorated were hospitalised.

Supplementary Table 2: Characteristics of the Wave 2 cohort.

	Overall (n = 17)	Peak severity mild or moderate (n = 9)	Peak severity severe or critical (n = 8)
**Age**			
**Median**	72.0	72.0	70.5
**(IQR)**	61.0-77.0	60.0-75.0	64.0-80.5
**Sex**			
**M**	10 (58.8%)	4 (44.4%)	6 (75.0%)
**F**	7 (41.2%)	5 (55.6%)	2 (25.0%)
**Ethnicity**			
**Asian**	11 (64.7%)	6 (66.7%)	5 (62.5%)
**White**	4 (23.5%)	2 (22.2%)	2 (25.0%)
**Black**	1 (5.9%)	0 (0.0%)	1 (12.5%)
**Other**	1 (5.9%)	1 (11.1%)	0 (0.0%)
**Diabetes**	11 (64.7%)	6 (66.7%)	5 (62.5%)
**Current smoker**	0 (0.0%)	0 (0.0%)	0 (0.0%)
**ESKD cause**			
**DN**	10 (58.8%)	6 (66.7%)	4 (50.0%)
**HTN/vascular**	0 (0.0%)	0 (0.0%)	0 (0.0%)
**GN/autoimmune**	1 (5.9%)	0 (0.0%)	1 (12.5%)
**Genetic**	0 (0.0%)	0 (0.0%)	0 (0.0%)
**Other/unknown**	6 (35.3%)	3 (33.3%)	3 (37.5%)
**Hospitalisation due to COVID-19**	9 (52.9%)	1 (11.1%)	8 (100%)
**Fatal COVID-19**	4 (23.5%)	0 (0.0%)	4 (50.0%)

DN = diabetic nephropathy. GN = glomerulonephritis. HTN = hypertension. IQR = inter-quartile range. Subsets defined according to peak WHO severity over the course of the illness.

Supplementary Table 3: Transcriptomic modules associated with disease trajectory.

Module code	Module name	Size	Selected enrichments	Primary cell type	Hub genes	Severity association
**tB**	Granulocyte cell-like module 1	1,309	R: Neutrophil degranulation<br>R: Innate immune system<br>R: ROS and RNS production in phagocytes<br>R: Oxidative stress-induced senescence	Neutrophils	*TECPR2, CSF3R, STX3, MMP25, BASP1, MBOAT7, NCF4, GLT1D1, RNF24, DHX34*	↑
**tJ**	Granulocyte cell-like module 2	169	R: Neutrophil degranulation<br>R: Innate immune system<br>R: Antimicrobial peptides<br>NABA: Matrisome	Macrophages (M0)	*CEACAM8, BPI, CD24, CEACAM6, ABCA13, DEFA4, LTF, AZU1, ELANE, LCN2*	↑*
**tL**	Plasma cell module	655	R: Cell cycle<br>R: MHC class II antigen presentation<br>WP: DNA damage response<br>R: Factors involved in megakaryocyte development and platelet production	Plasma cells	*RRM2, FOXM1, MKI67, TPX2, BUB1, KIFC1, TK1, MZB1, CDK1, CCNA2*	↑
**tP**	Nuclear and cell cycle module	464	None significant	B cells naive	*SNRPA1, CEP95, THUMPD2, CENPC, ZNF326, NDUFAF5, ILKAP, SUPV3L1, USP36, GOLT1B*	↓
**tC**	T-cell activity module 1	545	KEGG: Natural killer cell mediated cytotoxicity<br>R: Immunoregulatory interactions between a lymphoid and a non-lymphoid cell<br>PID: CD8 TCR downstream pathway	NK cells resting	*SAMD3, ADGRG1, ZAP70, PYHIN1, FCRL6, PRSS23, FGFBP2, NFATC2, PTCH1, LLGL2*	↓
**tF**	T-cell activity module 2	761	WP: T-cell receptor and co-stimulatory signaling	T cells CD4 memory resting	*PLCG1, LCK, UBASH3A, ABCD2, LINC00649, PRKCQ-AS1, TC2N, LINC01550, SEPTIN1, NLRC3*	↓
**tI**	Monocyte module	299	R: PD1 signaling<br>KEGG: Viral myocarditis<br>KEGG: Antigen processing and presentation<br>KEGG: Cell adhesion molecules<br>R: Interferon gamma signaling	Monocytes	*PLXNB2, NAAA, CSF1R, PSAP, CARD9, SLC7A7, PEA15, ARHGEF10L, ZNF385A, ARRB1*	↓
**tN**	Allergy-related module	54	WP: IL-3 signaling pathway<br>KEGG: Asthma	T cells CD4 memory resting	*HDC, LINC02458, CPA3, GATA2, AKAP12, MS4A2, ENPP3, FCER1A, TRIM51EP, SLC45A3*	↓

Transcriptomic modules associated with disease trajectory (i.e. with a significant TxCC interaction) are tabulated and assigned names that are representative of their members. Size = the number of genes assigned to the module. Primary cell type indicates the CIBERSORTx cell type with the greatest positive correlation with the module's eigengene. Severity association indicates whether the module's eigengene is positively (↑) or negatively (↓) associated with contemporaneous WHO severity (5% FDR, LMM).

* Module tJ was positively correlated with severity, but this association was not significant at 5% FDR.

R = Reactome. WP = WikiPathways. PID = Pathway Interaction Database.

Supplementary Table 4: Proteomic modules associated with disease trajectory.

Module code	Module name	Size	Selected enrichments	Hub genes	Severity association
**p12**	Histone-associated module	40	R: HDACs deacetylate histones<br>KEGG: Systemic lupus erythematosus<br>R: Chromatin modifying enzymes<br>R: HCMV late events	H2AC1, H2AW, H2BU1, H2BC21, H2BC12, H2AZ1, CELF2, H2AC11, EEF1B2, MMP17	↑
**p9**	Splicing and nuclear module 1	241	PID: FRA pathway<br>WP: Striated muscle contraction<br>R: Complement cascade<br>KEGG: Spliceosome	CLSTN3, KHSRP, OIT3, NELFA, EWSR1, CETP, PUF60, SRSF6, NXT1, ARHGAP36	↑
**p8**	Splicing and nuclear module 2	107	R: Processing of capped intron containing pre-mRNA<br>R: mRNA splicing<br>KEGG: Spliceosome	CILP, FUBP1, TSSC4, ALKAL2, STMN3, STMN1, STMN2, UBE2Z, MAFG, LEMD1	↑
**p1**	Mixed immune module 1	281	WP: Development of pulmonary dendritic cells and macrophage subsets	MRPL52, PLA2G2C, ITGAL, NLRP4, GDF3, VSTM4, ITGB1, SVBP, ANXA8, UBD	↑
**p7**	Mixed immune module 2	66	None significant	DEFB135, SPINK14, VAT1L, NT5E, PAXIP1, ZHX3, ODC1, PLEKHM2, DEFB112, F3	↓

Proteomic modules associated with disease trajectory (i.e. with a significant TxCC interaction) are tabulated and assigned names that are representative of their members. Size = the number of proteins assigned to the module. Severity association indicates whether the module’s eigenprotein is positively (↑) or negatively (↓) associated with contemporaneous WHO severity (5% FDR, LMM).
R = Reactome. WP = WikiPathways. PID = Pathway Interaction Database.

Supplementary Table 5. List of antibodies used in flow cytometry.

Antibodies	Source	Clone	Identifier	Dilutions
BV421-CD4	BioLegend	A161A1	357424	1/100
BV421-CD14	BioLegend	63D3	367144	1/100
PB-CD57	BioLegend	QA17A04	393316	1/100
PB-Foxp3 (Intracellular)	BioLegend	206D	320116	1/20
BV510-CD95	BioLegend	DX2	305640	1/100
BV510-IgD	BioLegend	IA6-2	348219	1/100
BV605-CCR7	BioLegend	G043H7	353224	1/100
BV605-CD4	BioLegend	SK3	344645	1/100
BV650-HLA-DR	BioLegend	L243	307650	1/20
BV650-CD123	BioLegend	6H6	306032	1/100
BV711-KLRG1	BioLegend	2F1/KLRG1	138427	1/100
BV711-CD11c	BioLegend	3.9	301630	1/100
BV750-CD45RA	BioLegend	HI100	304166	1/100
BV750-HLA-DR	BioLegend	L243	307672	1/20
BV785-CD19	BioLegend	HIB19	302240	1/100
AF488-CD3	BioLegend	HIT3a	300320	1/100
BB515-CD152/CTLA-4 (Intracellular)	BD Bioscience	BNI3	566917	1/50
BB515-CD8	BD Bioscience	RPA-T8	564526	1/100
PE-CD56	BioLegend	39D5	355504	1/100
PE-CD335	BioLegend	9^E2	331908	1/100
PE- CD16	BioLegend	3G8	302008	1/100
PE- CD14	BioLegend	63D3	367104	1/100
PE- CD19	BioLegend	HIB19	302208	1/100
PE-Siglec-1	BioLegend	7-239	346004	1/100
PEDAZ594-CD69	BioLegend	FN50	310941	1/100
PEDAZ594-NKG2D	BioLegend	1D11	320828	1/100
PE-Cy5-CD27	Thermofisher	O323	15-0279-42	1/100
PE-Cy5-CD25	Biolegend	BC96	302608	1/50
Percp-cy5.5-Ki67 (Intracellular)	BioLegend	Ki-67	350520	1/20
Percp-ef710-gdTCR	Thermofisher	B1.1	46-9959-42	1/50
Percp-ef710-CD16	Thermofisher	CB16	46-0168-42	1/100
PE-Cy7-PD1	BioLegend	A17188B	621616	1/100
PE-Cy7-CD141	Biolegend	M80	344110	1/100
AF647-CD45	BioLegend	HI30	304056	1/100
AF700-CD8	BioLegend	HIT8a	300920	1/100
AF700-CD66b	BioLegend	VI MA81	305114	1/100
APC/Fire750-CD38	BioLegend	HB-7	356626	1/100

Titles for Supplementary Data - (in Excel file)

Supplementary Data 1A. Differential gene expression analysis comparing COVID-19 positive versus negative PBMC samples. Contains the linear mixed model estimates and corresponding P-values for each gene, for both the Wave 1 and Wave 2 cohorts. The column Aggregated Score represents the RRA score for the P-values from both cohorts.

Supplementary Data 1B. Gene set analysis comparing COVID-19 positive versus negative samples. Contains the linear mixed model estimates and corresponding P-values for each GSVA gene set, for both the Wave 1 and Wave 2 cohorts. The column Aggregated Score represents the RRA score for the P-values from both cohorts.

Supplementary Data 1C. Protein annotations. List of proteins measured by the SomaScan v4.1 assay, their corresponding UniProt and GeneIDs, and their annotations in the human protein atlas.

Supplementary Data 1D. Differential plasma protein abundance analysis comparing COVID-19 positive versus negative samples. Contains the linear mixed model estimates and corresponding P-values for each protein, for both the Wave 1 and Wave 2 cohorts. The column Aggregated Score represents the RRA score for the P-values from both cohorts.

Supplementary Data 1E. Protein set analysis comparing COVID-19 positive versus negative samples. Contains the linear mixed model estimates and corresponding P-values for each GSVA protein set, for both the Wave 1 and Wave 2 cohorts. The column Aggregated Score represents the RRA score for the P-values from both cohorts.

Supplementary Data 1F. Transcriptomic associations with contemporaneous COVID-19 severity. Associations with 4-level ordinal WHO severity score at the time of the sample. Contains the linear mixed model estimates and corresponding P-values for each gene, for both the Wave 1 and Wave 2 cohorts. The column Aggregated Score represents the RRA score for the P-values from both cohorts.

Supplementary Data 1G. Associations of gene sets with contemporaneous COVID-19 severity. Associations with 4-level ordinal WHO severity score at the time of the sample. Contains the linear mixed model estimates and corresponding P-values for each GSVA gene set, for both the Wave 1 and Wave 2 cohorts. The column Aggregated Score represents the RRA score for the P-values from both cohorts.

Supplementary Data 1H. Proteomic associations with contemporaneous COVID-19 severity. Associations with 4-level ordinal WHO severity score at the time of the sample. Contains the linear mixed model estimates and corresponding P-values for each protein, for both the Wave 1 and Wave 2 cohorts. The column Aggregated Score represents the RRA score for the P-values from both cohorts.

Supplementary Data 1I. Associations of protein sets with contemporaneous COVID-19 severity. Associations with 4-level ordinal WHO severity score at the time of the sample. Contains the linear mixed model estimates and corresponding P-values for each GSVA gene set, for both the Wave 1 and Wave 2 cohorts. The column Aggregated Score represents the RRA score for the P-values from both cohorts.

Supplementary Data 1J. The membership of genes to WGCNA transcriptomic modules.

Supplementary Data 1K. The membership of proteins to WGCNA proteomic modules.

Supplementary Data 1L. Gene set enrichment of transcriptomic WGCNA modules. Overrepresentation analysis of gene sets for each module.

Supplementary Data 1M. Protein set enrichment of proteomic WGCNA modules. Overrepresentation analysis of protein sets for each module.

Supplementary Data 1N. Associations of imputed cell proportions with transcriptomic WGCNA modules. Contains the linear mixed model estimates and corresponding P-values for each WGCNA module – imputed cell type pair.

Supplementary Data 1O. Longitudinal profiles of cytokine proteins in plasma. P-values for the linear mixed modelling of cytokines and related proteins. P-values are included for the time, clinical course, and time * clinical course (TxCC) terms.

Supplementary Data 1P. Importance metrics for supervised learning of the transcriptome. The relative importance of genes according to the random forests (accuracy decrease) and lasso (number of models in which each gene had a non-zero coefficient during cross-validation) models. The metrics are normalised such that the most important gene has a value of 1.

Supplementary Data 1Q. Importance metrics for supervised learning of the proteome. The relative importance of proteins according to the random forests (accuracy decrease) and lasso (number of models in which each protein had a non-zero coefficient during cross-validation) models. The metrics are normalised such that the most important gene has a value of 1.

Supplementary Data 1R. Multi-omic supervised learning importance metrics. The relative importance of features (genes or proteins) according to the random forests (accuracy decrease) and lasso (number of models in which each feature had a non-zero coefficient during cross-validation) models. The metrics are normalised such that the most important gene has a value of 1.

Supplementary Data 1S. Paired differential expression analysis of pre-infection versus convalescent samples. Contains the linear mixed model estimates and corresponding P-values for each gene.

Supplementary Data 1T. Gene set enrichment of convalescence analysis. Contains the linear mixed model estimates and corresponding P-values for each GSVA gene set, for both the Wave 1 and Wave 2 cohorts. The column Aggregated Score represents the RRA score for the P-values from both cohorts.

Supplementary Data 1U. Potential ESKD-specific enrichments. Protein sets that were significant (FDR < 0.01) in our cohorts, but without any trend towards significance (unadjusted P > 0.05) in the data of Filbin *et al.*

Supplementary References

1. Ahern, D. J. *et al.* A blood atlas of COVID-19 defines hallmarks of disease severity and specificity. *Cell* **185**, 916-938.e58 (2022) doi:10.1016/j.cell.2022.01.012.

2. Filbin, M. R. *et al.* Longitudinal proteomic analysis of severe COVID-19 reveals survival-associated signatures, tissue-specific cell death, and cell-cell interactions. *Cell Reports Med.* **2**, 100287 (2021) doi:10.1016/j.xcrm.2021.100287.

3. Su, C.-Y. *et al.* Circulating proteins to predict adverse COVID-19 outcomes. *medRxiv* 2021.10.04.21264015 (2021) doi:10.1101/2021.10.04.21264015.
