# Supplementary material for: Multi-omics identify falling LRRC15 as a COVID-19 severity marker and persistent pro-thrombotic signals in convalescence
Source: Nat Commun. 2022 Dec 15;13:7775. doi: 10.1038/s41467-022-35454-4 (PMC9753891; doi:10.1038/s41467-022-35454-4)
Supplement: Supplementary file 3 — Description of Additional Supplementary Files [file 41467_2022_35454_MOESM3_ESM.pdf]

Description of Additional Supplementary Files

Supplementary Data 1A. Differential gene expression analysis comparing COVID-19 positive versus negative PBMC samples. Contains the linear mixed model estimates and corresponding P-values for each gene, for both the Wave 1 and Wave 2 cohorts. The column Aggregated Score represents the RRA score for the P-values from both cohorts.

Supplementary Data 1B. Gene set analysis comparing COVID-19 positive versus negative samples. Contains the linear mixed model estimates and corresponding P-values for each GSVA gene set, for both the Wave 1 and Wave 2 cohorts. The column Aggregated Score represents the RRA score for the P-values from both cohorts.

Supplementary Data 1C. Protein annotations. List of proteins measured by the SomaScan v4.1 assay, their corresponding UniProt and GeneIDs, and their annotations in the human protein atlas.

Supplementary Data 1D. Differential plasma protein abundance analysis comparing COVID-19 positive versus negative samples. Contains the linear mixed model estimates and corresponding P-values for each protein, for both the Wave 1 and Wave 2 cohorts. The column Aggregated Score represents the RRA score for the P-values from both cohorts.

Supplementary Data 1E. Protein set analysis comparing COVID-19 positive versus negative samples. Contains the linear mixed model estimates and corresponding P-values for each GSVA protein set, for both the Wave 1 and Wave 2 cohorts. The column Aggregated Score represents the RRA score for the P-values from both cohorts.

Supplementary Data 1F. Transcriptomic associations with contemporaneous COVID-19 severity. Associations with 4-level ordinal WHO severity score at the time of the sample. Contains the linear mixed model estimates and corresponding P-values for each gene, for both the Wave 1 and Wave 2 cohorts. The column Aggregated Score represents the RRA score for the P-values from both cohorts.

Supplementary Data 1G. Associations of gene sets with contemporaneous COVID-19 severity. Associations with 4-level ordinal WHO severity score at the time of the sample. Contains the linear mixed model estimates and corresponding P-values for each GSVA gene set, for both the Wave 1 and Wave 2 cohorts. The column Aggregated Score represents the RRA score for the P-values from both cohorts.

Supplementary Data 1H. Proteomic associations with contemporaneous COVID-19 severity. Associations with 4-level ordinal WHO severity score at the time of the sample. Contains the linear mixed model estimates and corresponding P-values for each protein, for both the Wave 1 and Wave 2 cohorts. The column Aggregated Score represents the RRA score for the P-values from both cohorts.

Supplementary Data 1I. Associations of protein sets with contemporaneous COVID-19 severity. Associations with 4-level ordinal WHO severity score at the time of the sample. Contains the linear mixed model estimates and corresponding P-values for each GSVA gene set, for both the Wave 1 and Wave 2 cohorts. The column Aggregated Score represents the RRA score for the P-values from both cohorts.

Supplementary Data 1J. The membership of genes to WGCNA transcriptomic modules.

Supplementary Data 1K. The membership of proteins to WGCNA proteomic modules.

Supplementary Data 1L. Gene set enrichment of transcriptomic WGCNA modules. Overrepresentation analysis of gene sets for each module.

Supplementary Data 1M. Protein set enrichment of proteomic WGCNA modules. Overrepresentation analysis of protein sets for each module.

Supplementary Data 1N. Associations of imputed cell proportions with transcriptomic WGCNA modules. Contains the linear mixed model estimates and corresponding P-values for each WGCNA module – imputed cell type pair.

Supplementary Data 1O. Longitudinal profiles of cytokine proteins in plasma. P-values for the linear mixed modelling of cytokines and related proteins. P-values are included for the time, clinical course, and time * clinical course (TxCC) terms.

Supplementary Data 1P. Importance metrics for supervised learning of the transcriptome. The relative importance of genes according to the random forests (accuracy decrease) and lasso (number of models in which each gene had a non-zero coefficient during cross-validation) models. The metrics are normalised such that the most important gene has a value of 1.

Supplementary Data 1Q. Importance metrics for supervised learning of the proteome. The relative importance of proteins according to the random forests (accuracy decrease) and lasso (number of models in which each protein had a non-zero coefficient during cross-validation) models. The metrics are normalised such that the most important gene has a value of 1.

Supplementary Data 1R. Multi-omic supervised learning importance metrics. The relative importance of features (genes or proteins) according to the random forests (accuracy decrease) and lasso (number of models in which each feature had a non-zero coefficient during cross-validation) models. The metrics are normalised such that the most important gene has a value of 1.

Supplementary Data 1S. Paired differential expression analysis of pre-infection versus convalescent samples. Contains the linear mixed model estimates and corresponding P-values for each gene.

Supplementary Data 1T. Gene set enrichment of convalescence analysis. Contains the linear mixed model estimates and corresponding P-values for each GSVA gene set, for both the Wave 1 and Wave 2 cohorts. The column Aggregated Score represents the RRA score for the P-values from both cohorts.

Supplementary Data 1U. Potential ESKD-specific enrichments. Protein sets that were significant (FDR < 0.01) in our cohorts, but without any trend towards significance (unadjusted P > 0.05) in the data of Filbin *et al.*
