# Supplementary material for: Multi-omics identify falling LRRC15 as a COVID-19 severity marker and persistent pro-thrombotic signals in convalescence
Source: Nat Commun. 2022 Dec 15;13:7775. doi: 10.1038/s41467-022-35454-4 (PMC9753891; doi:10.1038/s41467-022-35454-4)
Supplement: Supplementary file 5 — Reporting Summary [file 41467_2022_35454_MOESM5_ESM.pdf]

nature portfolio

Corresponding author(s): David Thomas and James Peters

Last updated by author(s): Nov 23, 2022

Reporting Summary

Nature Portfolio wishes to improve the reproducibility of the work that we publish. This form provides structure for consistency and transparency in reporting. For further information on Nature Portfolio policies, see our Editorial Policies and the Editorial Policy Checklist.

Statistics

For all statistical analyses, confirm that the following items are present in the figure legend, table legend, main text, or Methods section.

n/a	Confirmed	
☐	☒	The exact sample size (*n*) for each experimental group/condition, given as a discrete number and unit of measurement
☐	☒	A statement on whether measurements were taken from distinct samples or whether the same sample was measured repeatedly
☐	☒	The statistical test(s) used AND whether they are one- or two-sided<br>*Only common tests should be described solely by name; describe more complex techniques in the Methods section.*
☐	☒	A description of all covariates tested
☐	☒	A description of any assumptions or corrections, such as tests of normality and adjustment for multiple comparisons
☐	☒	A full description of the statistical parameters including central tendency (e.g. means) or other basic estimates (e.g. regression coefficient) AND variation (e.g. standard deviation) or associated estimates of uncertainty (e.g. confidence intervals)
☐	☒	For null hypothesis testing, the test statistic (e.g. *F*, *t*, *r*) with confidence intervals, effect sizes, degrees of freedom and *P* value noted<br>*Give P values as exact values whenever suitable.*
☒	☐	For Bayesian analysis, information on the choice of priors and Markov chain Monte Carlo settings
☒	☐	For hierarchical and complex designs, identification of the appropriate level for tests and full reporting of outcomes
☐	☒	Estimates of effect sizes (e.g. Cohen's *d*, Pearson's *r*), indicating how they were calculated

Our web collection on statistics for biologists contains articles on many of the points above.

Software and code

Policy information about availability of computer code

Data collection: Flow cytometry data was collected using Aurora Spectral Flow Cytometry (Cytek®)

Data analysis: As described in the code availability statement, custom code has been published on Zenodo: https://doi.org/10.5281/zenodo.7333789

FlowJo software, version 10 (Tree Star Inc. Ashland, OR, USA) were used for analysis of all samples by flow cytometry. GraphPad Prism version 9 was used for downstream statistical analysis.

RNA-seq data was processed using Nextflow's nf-core RNA-seq pipeline (v3.2).

Proteomic data was extracted from SomaLogic's .adat format using the R package SomaDataIO (v5.3.0).

See also the output of R's sessionInfo() function below, which describes all software packages loaded for each analysis of the transcriptomic and proteomic data:

Differential expression/abundance analysis
```
## R version 4.1.2 (2021-11-01)
## Platform: x86_64-w64-mingw32/x64 (64-bit)
## Running under: Windows 10 x64 (build 19044)
##
## Matrix products: default
##
```

```
## locale:
## [1] LC_COLLATE=English_United Kingdom.1252
## [2] LC_CTYPE=English_United Kingdom.1252
## [3] LC_MONETARY=English_United Kingdom.1252
## [4] LC_NUMERIC=C
## [5] LC_TIME=English_United Kingdom.1252
##
## attached base packages:
## [1] stats4    stats     graphics  grDevices utils     datasets  methods
## [8] base
##
## other attached packages:
##  [1] ggpubr_0.4.0                biomaRt_2.50.3
##  [3] GSVA_1.42.0                 lmerTest_3.1-3
##  [5] lme4_1.1-28                 Matrix_1.3-4
##  [7] edgeR_3.36.0                variancePartition_1.24.0
##  [9] BiocParallel_1.28.3         limma_3.50.1
## [11] ggplot2_3.3.5               SummarizedExperiment_1.24.0
## [13] Biobase_2.54.0              GenomicRanges_1.46.1
## [15] GenomeInfoDb_1.30.1         IRanges_2.28.0
## [17] S4Vectors_0.32.3            BiocGenerics_0.40.0
## [19] MatrixGenerics_1.6.0        matrixStats_0.61.0
## [21] data.table_1.14.2
##
## loaded via a namespace (and not attached):
##   [1] minqa_1.2.4                colorspace_2.0-2
##   [3] ggsignif_0.6.3             ellipsis_0.3.2
##   [5] XVector_0.34.0             rstudioapi_0.13
##   [7] bit64_4.0.5                AnnotationDbi_1.56.2
##   [9] fansi_1.0.2                xml2_1.3.3
##  [11] codetools_0.2-18           splines_4.1.2
##  [13] sparseMatrixStats_1.6.0    doParallel_1.0.17
##  [15] cachem_1.0.6               knitr_1.37
##  [17] nloptr_2.0.0               pbkrtest_0.5.1
##  [19] broom_0.7.12               annotate_1.72.0
##  [21] dbplyr_2.1.1               png_0.1-7
##  [23] graph_1.72.0               HDF5Array_1.22.1
##  [25] compiler_4.1.2             httr_1.4.2
##  [27] backports_1.4.1            assertthat_0.2.1
##  [29] fastmap_1.1.0              cli_3.1.1
##  [31] BiocSingular_1.10.0        htmltools_0.5.2
##  [33] prettyunits_1.1.1          tools_4.1.2
##  [35] rsvd_1.0.5                 gtable_0.3.0
##  [37] glue_1.6.1                 GenomeInfoDbData_1.2.7
##  [39] reshape2_1.4.4             dplyr_1.0.8
##  [41] rappdirs_0.3.3             Rcpp_1.0.8
##  [43] carData_3.0-5              vctrs_0.3.8
##  [45] Biostrings_2.62.0          rhdf5filters_1.6.0
##  [47] nlme_3.1-153               DelayedMatrixStats_1.16.0
##  [49] iterators_1.0.14           xfun_0.29
##  [51] stringr_1.4.0              beachmat_2.10.0
##  [53] irlba_2.3.5                lifecycle_1.0.1
##  [55] gtools_3.9.2               rstatix_0.7.0
##  [57] XML_3.99-0.8               zlibbioc_1.40.0
##  [59] MASS_7.3-54                scales_1.1.1
##  [61] hms_1.1.1                  parallel_4.1.2
##  [63] rhdf5_2.38.0               curl_4.3.2
##  [65] SingleCellExperiment_1.16.0 yaml_2.2.2
##  [67] memoise_2.0.1              stringi_1.7.6
##  [69] RSQLite_2.2.10             ScaledMatrix_1.2.0
##  [71] foreach_1.5.2              filelock_1.0.2
##  [73] caTools_1.18.2             boot_1.3-28
##  [75] rlang_1.0.1                pkgconfig_2.0.3
##  [77] bitops_1.0-7               evaluate_0.15
##  [79] lattice_0.20-45            Rhdf5lib_1.16.0
##  [81] purrr_0.3.4                bit_4.0.4
##  [83] tidyselect_1.1.1           GSEABase_1.56.0
##  [85] plyr_1.8.6                 magrittr_2.0.2
##  [87] R6_2.5.1                   snow_0.4-4
##  [89] gplots_3.1.1               generics_0.1.2
##  [91] DelayedArray_0.20.0        DBI_1.1.2
##  [93] pillar_1.7.0               withr_2.4.3
##  [95] abind_1.4-5                KEGGREST_1.34.0
##  [97] RCurl_1.98-1.6             tibble_3.1.6
##  [99] car_3.0-12                 crayon_1.5.0
## [101] KernSmooth_2.23-20         utf8_1.2.2
```

```
## [103] BiocFileCache_2.2.1        rmarkdown_2.11
## [105] progress_1.2.2             locfit_1.5-9.4
## [107] grid_4.1.2                 blob_1.2.2
## [109] digest_0.6.29              xtable_1.8-4
## [111] tidyr_1.2.0                numDeriv_2016.8-1.1
## [113] munsell_0.5.0
```

GSVA analysis

```
## R version 4.1.2 (2021-11-01)
## Platform: x86_64-w64-mingw32/x64 (64-bit)
## Running under: Windows 10 x64 (build 19044)
##
## Matrix products: default
##
## locale:
## [1] LC_COLLATE=English_United Kingdom.1252
## [2] LC_CTYPE=English_United Kingdom.1252
## [3] LC_MONETARY=English_United Kingdom.1252
## [4] LC_NUMERIC=C
## [5] LC_TIME=English_United Kingdom.1252
##
## attached base packages:
## [1] stats4    stats     graphics  grDevices utils     datasets  methods
## [8] base
##
## other attached packages:
##  [1] ggpubr_0.4.0                ggplot2_3.3.5
##  [3] edgeR_3.36.0                limma_3.50.1
##  [5] biomaRt_2.50.3              GSVA_1.42.0
##  [7] lmerTest_3.1-3              lme4_1.1-28
##  [9] Matrix_1.3-4                SummarizedExperiment_1.24.0
## [11] Biobase_2.54.0              GenomicRanges_1.46.1
## [13] GenomeInfoDb_1.30.1         IRanges_2.28.0
## [15] S4Vectors_0.32.3            BiocGenerics_0.40.0
## [17] MatrixGenerics_1.6.0        matrixStats_0.61.0
## [19] data.table_1.14.2
##
## loaded via a namespace (and not attached):
##   [1] minqa_1.2.4                colorspace_2.0-2
##   [3] ggsignif_0.6.3             ellipsis_0.3.2
##   [5] XVector_0.34.0             rstudioapi_0.13
##   [7] bit64_4.0.5                AnnotationDbi_1.56.2
##   [9] fansi_1.0.2                xml2_1.3.3
##  [11] splines_4.1.2              sparseMatrixStats_1.6.0
##  [13] cachem_1.0.6               knitr_1.37
##  [15] nloptr_2.0.0               broom_0.7.12
##  [17] annotate_1.72.0            dbplyr_2.1.1
##  [19] png_0.1-7                  graph_1.72.0
##  [21] HDF5Array_1.22.1           compiler_4.1.2
##  [23] httr_1.4.2                 backports_1.4.1
##  [25] assertthat_0.2.1           fastmap_1.1.0
##  [27] cli_3.1.1                  BiocSingular_1.10.0
##  [29] htmltools_0.5.2            prettyunits_1.1.1
##  [31] tools_4.1.2                rsvd_1.0.5
##  [33] gtable_0.3.0               glue_1.6.1
##  [35] GenomeInfoDbData_1.2.7     dplyr_1.0.8
##  [37] rappdirs_0.3.3             Rcpp_1.0.8
##  [39] carData_3.0-5              vctrs_0.3.8
##  [41] Biostrings_2.62.0          rhdf5filters_1.6.0
##  [43] nlme_3.1-153               DelayedMatrixStats_1.16.0
##  [45] xfun_0.29                  stringr_1.4.0
##  [47] beachmat_2.10.0            lifecycle_1.0.1
##  [49] irlba_2.3.5                rstatix_0.7.0
##  [51] XML_3.99-0.8               zlibbioc_1.40.0
##  [53] MASS_7.3-54                scales_1.1.1
##  [55] hms_1.1.1                  parallel_4.1.2
##  [57] rhdf5_2.38.0               SingleCellExperiment_1.16.0
##  [59] yaml_2.2.2                 curl_4.3.2
##  [61] memoise_2.0.1              stringi_1.7.6
##  [63] RSQLite_2.2.10             ScaledMatrix_1.2.0
##  [65] filelock_1.0.2             boot_1.3-28
##  [67] BiocParallel_1.28.3        rlang_1.0.1
##  [69] pkgconfig_2.0.3            bitops_1.0-7
##  [71] evaluate_0.15              lattice_0.20-45
##  [73] purrr_0.3.4                Rhdf5lib_1.16.0
##  [75] bit_4.0.4                  tidyselect_1.1.1
```

```
## [77] GSEABase_1.56.0           magrittr_2.0.2
## [79] R6_2.5.1                  generics_0.1.2
## [81] DelayedArray_0.20.0       DBI_1.1.2
## [83] pillar_1.7.0              withr_2.4.3
## [85] abind_1.4-5               KEGGREST_1.34.0
## [87] RCurl_1.98-1.6            tibble_3.1.6
## [89] car_3.0-12                crayon_1.5.0
## [91] utf8_1.2.2                BiocFileCache_2.2.1
## [93] rmarkdown_2.11            progress_1.2.2
## [95] locfit_1.5-9.4            grid_4.1.2
## [97] blob_1.2.2                digest_0.6.29
## [99] xtable_1.8-4              tidyr_1.2.0
## [101] numDeriv_2016.8-1.1      munsell_0.5.0
```

Supervised learning

```
## R version 4.1.2 (2021-11-01)
## Platform: x86_64-w64-mingw32/x64 (64-bit)
## Running under: Windows 10 x64 (build 19044)
##
## Matrix products: default
##
## locale:
## [1] LC_COLLATE=English_United Kingdom.1252
## [2] LC_CTYPE=English_United Kingdom.1252
## [3] LC_MONETARY=English_United Kingdom.1252
## [4] LC_NUMERIC=C
## [5] LC_TIME=English_United Kingdom.1252
##
## attached base packages:
## [1] stats4    stats     graphics  grDevices utils     datasets  methods
## [8] base
##
## other attached packages:
##  [1] ggpubr_0.4.0                rmcorr_0.4.5
##  [3] biomaRt_2.50.3              caret_6.0-90
##  [5] lattice_0.20-45             ggplot2_3.3.5
##  [7] DESeq2_1.34.0               edgeR_3.36.0
##  [9] limma_3.50.1                data.table_1.14.2
## [11] SummarizedExperiment_1.24.0 Biobase_2.54.0
## [13] GenomicRanges_1.46.1        GenomeInfoDb_1.30.1
## [15] IRanges_2.28.0              S4Vectors_0.32.3
## [17] BiocGenerics_0.40.0         MatrixGenerics_1.6.0
## [19] matrixStats_0.61.0
##
## loaded via a namespace (and not attached):
##   [1] backports_1.4.1        BiocFileCache_2.2.1    plyr_1.8.6
##   [4] splines_4.1.2          BiocParallel_1.28.3    listenv_0.8.0
##   [7] digest_0.6.29          foreach_1.5.2          htmltools_0.5.2
##  [10] fansi_1.0.2            magrittr_2.0.2         memoise_2.0.1
##  [13] recipes_0.2.0          globals_0.14.0         Biostrings_2.62.0
##  [16] annotate_1.72.0        gower_1.0.0            hardhat_0.2.0
##  [19] prettyunits_1.1.1      colorspace_2.0-2       blob_1.2.2
##  [22] rappdirs_0.3.3         xfun_0.29              dplyr_1.0.8
##  [25] crayon_1.5.0           RCurl_1.98-1.6         genefilter_1.76.0
##  [28] survival_3.2-13        iterators_1.0.14       glue_1.6.1
##  [31] gtable_0.3.0           ipred_0.9-12           zlibbioc_1.40.0
##  [34] XVector_0.34.0         DelayedArray_0.20.0    car_3.0-12
##  [37] future.apply_1.8.1     shape_1.4.6            abind_1.4-5
##  [40] scales_1.1.1           DBI_1.1.2              rstatix_0.7.0
##  [43] Rcpp_1.0.8             xtable_1.8-4           progress_1.2.2
##  [46] bit_4.0.4              lava_1.6.10            prodlim_2019.11.13
##  [49] glmnet_4.1-3           httr_1.4.2             RColorBrewer_1.1-2
##  [52] ellipsis_0.3.2         pkgconfig_2.0.3        XML_3.99-0.8
##  [55] farver_2.1.0           nnet_7.3-16            dbplyr_2.1.1
##  [58] locfit_1.5-9.4         utf8_1.2.2             tidyselect_1.1.1
##  [61] labeling_0.4.2         rlang_1.0.1            reshape2_1.4.4
##  [64] AnnotationDbi_1.56.2   munsell_0.5.0          tools_4.1.2
##  [67] cachem_1.0.6           cli_3.1.1              generics_0.1.2
##  [70] RSQLite_2.2.10         broom_0.7.12           evaluate_0.15
##  [73] stringr_1.4.0          fastmap_1.1.0          yaml_2.2.2
##  [76] ModelMetrics_1.2.2.2   knitr_1.37             bit64_4.0.5
##  [79] purrr_0.3.4            randomForest_4.7-1     KEGGREST_1.34.0
##  [82] future_1.24.0          nlme_3.1-153           xml2_1.3.3
##  [85] compiler_4.1.2         rstudioapi_0.13        filelock_1.0.2
##  [88] curl_4.3.2             png_0.1-7              ggsignif_0.6.3
##  [91] tibble_3.1.6           geneplotter_1.72.0     stringi_1.7.6
```

```
## [94] highr_0.9 Matrix_1.3-4 vctrs_0.3.8
## [97] pillar_1.7.0 lifecycle_1.0.1 bitops_1.0-7
## [100] R6_2.5.1 parallelly_1.30.0 codetools_0.2-18
## [103] MASS_7.3-54 assertthat_0.2.1 withr_2.4.3
## [106] GenomeInfoDbData_1.2.7 parallel_4.1.2 hms_1.1.1
## [109] grid_4.1.2 rpart_4.1-15 timeDate_3043.102
## [112] tidyr_1.2.0 class_7.3-19 rmarkdown_2.11
## [115] carData_3.0-5 pROC_1.18.0 lubridate_1.8.0
```

Modular analysis

```
## R version 4.1.2 (2021-11-01)
## Platform: x86_64-w64-mingw32/x64 (64-bit)
## Running under: Windows 10 x64 (build 19044)
##
## Matrix products: default
##
## locale:
## [1] LC_COLLATE=English_United Kingdom.1252
## [2] LC_CTYPE=English_United Kingdom.1252
## [3] LC_MONETARY=English_United Kingdom.1252
## [4] LC_NUMERIC=C
## [5] LC_TIME=English_United Kingdom.1252
##
## attached base packages:
## [1] splines stats4 parallel stats graphics grDevices utils
## [8] datasets methods base
##
## other attached packages:
## [1] ggpubr_0.4.0 ggplot2_3.3.5
## [3] ggeffects_1.1.1 lmerTest_3.1-3
## [5] lme4_1.1-28 Matrix_1.3-4
## [7] WGCNA_1.70-3 fastcluster_1.2.3
## [9] dynamicTreeCut_1.63-1 SummarizedExperiment_1.24.0
## [11] Biobase_2.54.0 GenomicRanges_1.46.1
## [13] GenomeInfoDb_1.30.1 IRanges_2.28.0
## [15] S4Vectors_0.32.3 BiocGenerics_0.40.0
## [17] MatrixGenerics_1.6.0 matrixStats_0.61.0
## [19] doParallel_1.0.17 iterators_1.0.14
## [21] foreach_1.5.2 rmcorr_0.4.5
##
## loaded via a namespace (and not attached):
## [1] minqa_1.2.4 colorspace_2.0-2 ggsignif_0.6.3
## [4] ellipsis_0.3.2 sjlabelled_1.1.8 estimability_1.3
## [7] htmlTable_2.4.0 XVector_0.34.0 base64enc_0.1-3
## [10] rstudioapi_0.13 farver_2.1.0 bit64_4.0.5
## [13] mvtnorm_1.1-3 AnnotationDbi_1.56.2 fansi_1.0.2
## [16] codetools_0.2-18 cachem_1.0.6 impute_1.68.0
## [19] knitr_1.37 Formula_1.2-4 nloptr_2.0.0
## [22] broom_0.7.12 cluster_2.1.2 GO.db_3.14.0
## [25] png_0.1-7 compiler_4.1.2 httr_1.4.2
## [28] emmeans_1.7.2 backports_1.4.1 assertthat_0.2.1
## [31] fastmap_1.1.0 cli_3.1.1 htmltools_0.5.2
## [34] tools_4.1.2 gtable_0.3.0 glue_1.6.1
## [37] GenomeInfoDbData_1.2.7 reshape2_1.4.4 dplyr_1.0.8
## [40] Rcpp_1.0.8 carData_3.0-5 vctrs_0.3.8
## [43] Biostrings_2.62.0 preprocessCore_1.56.0 nlme_3.1-153
## [46] insight_0.16.0 xfun_0.29 stringr_1.4.0
## [49] lifecycle_1.0.1 rstatix_0.7.0 zlibbioc_1.40.0
## [52] MASS_7.3-54 scales_1.1.1 RColorBrewer_1.1-2
## [55] yaml_2.2.2 memoise_2.0.1 gridExtra_2.3
## [58] rpart_4.1-15 latticeExtra_0.6-29 stringi_1.7.6
## [61] RSQLite_2.2.10 highr_0.9 checkmate_2.0.0
## [64] boot_1.3-28 rlang_1.0.1 pkgconfig_2.0.3
## [67] bitops_1.0-7 evaluate_0.15 lattice_0.20-45
## [70] purrr_0.3.4 labeling_0.4.2 htmlwidgets_1.5.4
## [73] bit_4.0.4 tidyselect_1.1.1 plyr_1.8.6
## [76] magrittr_2.0.2 R6_2.5.1 generics_0.1.2
## [79] Hmisc_4.6-0 DelayedArray_0.20.0 DBI_1.1.2
## [82] pillar_1.7.0 foreign_0.8-81 withr_2.4.3
## [85] survival_3.2-13 KEGGREST_1.34.0 abind_1.4-5
## [88] RCurl_1.98-1.6 nnet_7.3-16 tibble_3.1.6
## [91] crayon_1.5.0 car_3.0-12 utf8_1.2.2
## [94] rmarkdown_2.11 jpeg_0.1-9 grid_4.1.2
## [97] data.table_1.14.2 blob_1.2.2 flashClust_1.01-2
## [100] digest_0.6.29 xtable_1.8-4 tidyr_1.2.0
## [103] numDeriv_2016.8-1.1 munsell_0.5.0
```

For manuscripts utilizing custom algorithms or software that are central to the research but not yet described in published literature, software must be made available to editors and reviewers. We strongly encourage code deposition in a community repository (e.g. GitHub). See the Nature Portfolio guidelines for submitting code & software for further information.

Data

Policy information about availability of data

All manuscripts must include a data availability statement. This statement should provide the following information, where applicable:

- Accession codes, unique identifiers, or web links for publicly available datasets
- A description of any restrictions on data availability
- For clinical datasets or third party data, please ensure that the statement adheres to our policy

The individual-level transcriptomics (counts), proteomics and flow cytometry data are available without restriction from Zenodo (https://doi.org/10.5281/zenodo.6497251). Processed subsets of these data are provided in the Source Data files. The raw RNA-seq reads are available under restricted access to comply with UK GDPR legislation and can be obtained from the European Phenome-Genome archive (EGA) under study accession EGAS00001006778 (https://ega-archive.org/studies/EGAS00001006778).

In this study, we made use of the whole blood bulk RNA-seq generated by the COvid-19 Multi-omics Blood ATlas (COMBAT) Consortium study [7], which is available from Zenodo (https://doi.org/10.5281/zenodo.6120249). We also incorporated the SomaScan proteomics data of Filbin et al. [12], deposited in Mendeley Data (https://doi.org/10.17632/nf853r8xsj.2).

Human research participants

Policy information about studies involving human research participants and Sex and Gender in Research.

Reporting on sex and gender	Both male and female participants included, as described in Supplementary Tables 1 and 2. Individual-level sex data is available from Zenodo (https://doi.org/10.5281/zenodo.6497251).
Population characteristics	Characteristics are included in both the Supplementary Tables (Supplementary Tables 1, 2) and the individual-level data (https://doi.org/10.5281/zenodo.6497251).
Recruitment	Adult participants (18 years or over) were recruited from the Imperial College Renal and Transplant Centre and its satellite dialysis units, London, United Kingdom.<br><br>We recruited two cohorts of ESKD patients with COVID-19 (Figure 1A). All patients were receiving haemodialysis prior to acquiring COVID-19. The first cohort (Wave 1) were recruited during the initial phase of the COVID-19 pandemic (April-May 2020). Blood samples were taken from 53 patients with COVID-19 (Supplementary Table 1). Serial blood sampling was carried out where feasible (Figure 1B), given the pressure on hospital services and the effects of national lockdown. We also contemporaneously recruited 59 non-infected haemodialysis patients to provide a control group, selected to mirror the age, sex and ethnicity distribution of the COVID-19 cases (Supplementary Figure 1A-C).<br><br>The Wave 2 cohort consisted of 17 ESKD patients with COVID-19 infected during the resurgence of cases in January-March 2021 (Supplementary Table 2). These 17 individuals had all been recruited as part of the COVID-19 negative control group during Wave 1, and so a pre-infection sample collected in April/May 2020 (8-9 months preceding infection) was also available. For the Wave 2 cohort, we systematically acquired serial samples for all patients at regular intervals (every 2-3 days over the course of the acute illness) (Figure 1C). Additionally, for 12 of these 17 patients, we acquired convalescent samples at approximately 2 months post the acute COVID-19 episode (range 41-55 days from the initial sample). Convalescent samples were unavailable for four patients who died and for one patient due to logistical difficulties in sample collection.<br><br>To minimise variation related to the timing of dialysis, blood samples were taken prior to commencing a haemodialysis session.<br><br>Potential recruitment bias:<br>The demographics of our study cohort are broadly similar to our local ESKD population. Patients who presented to hospital with decreased levels of consciousness would not have been able to consent. Nevertheless, we had representation of patients with critical and fatal disease.
Ethics oversight	Study ethics were reviewed by the UK National Health Service (NHS) Health Research Authority (HRA) and Health and Care Research Wales (HCRW) Research Ethics Committee (reference 20/WA/0123: The impact of COVID-19 on patients with renal disease and immunosuppressed patients). Ethical approval was given. Study volunteers provided informed consent and did not receive financial or other compensation for participating in the study.

Note that full information on the approval of the study protocol must also be provided in the manuscript.

Field-specific reporting

Please select the one below that is the best fit for your research. If you are not sure, read the appropriate sections before making your selection.

☒ Life sciences ☐ Behavioural & social sciences ☐ Ecological, evolutionary & environmental sciences

For a reference copy of the document with all sections, see nature.com/documents/nr-reporting-summary-flat.pdf

Life sciences study design

All studies must disclose on these points even when the disclosure is negative.

Sample size

No formal power calculations were performed. Samples were obtained where feasible given the emergency healthy situation during the peaks of COVID-19 and the extreme pressure on the UK NHS.

Data exclusions

One sample was excluded from the proteomics data because the sample did not pass SomaLogic QC. Twelve samples with low cell number recovery (less than 10,000 PBMCs) were excluded from the flow cytometry analysis.

Replication

We present data from 2 cohorts, enabling assessment of findings that replicate in both. Both cohorts were from the same centre, so we lacked a truly independent external validation cohort. However, the differential expression gene expression and protein abundance analyses were performed separately for the Wave 1 and Wave 2 cohorts that were recruited approximately a year apart. Thus, we are able to examine how our findings replicate in two temporally distinct subcohorts, albeit with patients recruited from and samples processed in the same centre. To identify the genes that were consistently differentially expressed across both cohorts, we used robust rank aggregation (RRA) (Supplementary File 1A). We also present Venn diagrams (Supplementary Figures 25-26) that shows the overlap in the significant findings in the Wave 1 and Wave 2 analyses at 1% FDR for each analysis (and also the overlap with external data from non-ESKD COVID-19 patient cohorts).

For the PBMC transcriptomic analyses (Supplementary Figure 25), we observed the following:

• COVID-19 positive versus negative comparison:
3,468 genes significantly differentially expressed in both Wave 1 and 2 cohorts.
• For associations with COVID-19 severity (encoded as a 4-level ordinal variable):
363 genes significantly associated with severity in both Waves 1 and 2.

For the plasma proteomic analyses (Supplementary Figure 26):
• COVID-19 positive versus negative comparison:
730 proteins significantly differentially abundant in both Waves 1 and 2.
• For associations with COVID-19 severity:
98 proteins associated with severity in both Waves 1 and 2.

It should be noted that requiring FDR <0.01 represents a more stringent definition of replication than in used in most studies (which typically limit multiple testing burden in the validation cohort by only testing candidates that were significant in the primary cohort, and often use FDR <0.05). It should also be noted that this approach of intersecting lists of significant features will underestimate agreement between studies due to the use of a hard significance threshold; for example, a gene with a similar effect estimates in the discovery and validation cohorts may fall on opposite sides of the significance threshold and thus "fail to replicate" despite consistent findings. For a more detailed and technical explanation see also PMID 17428330 (Blangiardo, M. & Richardson, S. Statistical tools for synthesizing lists of differentially expressed features in related experiments. Genome Biol. 8, R54 (2007) doi:10.1186/gb-2007-8-4-r54).

For this reason, for each analysis we also provided plots and Pearson correlations of the estimated effect sizes for each gene (or protein) in the Wave 1 cohort versus Wave 2 cohort (Supplementary Figure 3A and a similar plot for proteins in Supplementary Figure 3B). This approach enables an assessment of consistency that is not dependent on the specific statistical threshold used. The correlation of effect sizes between Wave 1 and 2 were highly concordant for the COVID-19 positive versus negative differential gene expression analysis (Pearson's r 0.80). They were correlated, albeit less so far, for the differential proteomic abundant analysis (Pearson's r 0.57).

We also performed supervised learning using a train/test split. We are thus able to provide metrics of model performance on completely unseen data. These metrics are provided in the Supplementary Material (p. 7) for each supervised learning method (random forests or lasso) and data type (RNA-seq or plasma proteomics), as well as the ensembled model. In brief, the AUC for the ensembled model was 0.89 i.e. the model performs well.

Randomization

N/A: observational study.

Blinding

Analysts were not blinded to COVID-19 status or severity.

Reporting for specific materials, systems and methods

We require information from authors about some types of materials, experimental systems and methods used in many studies. Here, indicate whether each material, system or method listed is relevant to your study. If you are not sure if a list item applies to your research, read the appropriate section before selecting a response.

Materials & experimental systems

n/a	Involved in the study
☐	☒ Antibodies
☒	☐ Eukaryotic cell lines
☒	☐ Palaeontology and archaeology
☒	☐ Animals and other organisms
☐	☒ Clinical data
☒	☐ Dual use research of concern

Methods

n/a	Involved in the study
☒	☐ ChIP-seq
☐	☒ Flow cytometry
☒	☐ MRI-based neuroimaging

Antibodies

Antibodies used

Antibodies Source Clone Identifier Dilutions
BV421-CD4 BioLegend A161A1 357424 1/100
BV421-CD14 BioLegend 63D3 367144 1/100
PB-CD57 BioLegend QA17A04 393316 1/100
PB-Foxp3 (Intracellular) BioLegend 206D 320116 1/20
BV510-CD95 BioLegend DX2 305640 1/100
BV510-IgD BioLegend IA6-2 348219 1/100
BV605-CCR7 BioLegend G043H7 353224 1/100
BV605-CD4 BioLegend SK3 344645 1/100
BV650-HLA-DR BioLegend L243 307650 1/20
BV650-CD123 BioLegend 6H6 306032 1/100
BV711-KLRG1 BioLegend 2F1/KLRG1 138427 1/100
BV711-CD11c BioLegend 3.9 301630 1/100
BV750-CD45RA BioLegend HI100 304166 1/100
BV750-HLA-DR BioLegend L243 307672 1/20
BV785-CD19 BioLegend HIB19 302240 1/100
AF488-CD3 BioLegend HIT3a 300320 1/100
BB515-CD152/CTLA-4 (Intracellular) BD Bioscience BNI3 566917 1/50
BB515-CD8 BD Bioscience RPA-T8 564526 1/100
PE-CD56 BioLegend 39D5 355504 1/100
PE-CD335 BioLegend 9^E2 331908 1/100
PE- CD16 BioLegend 3G8 302008 1/100
PE- CD14 BioLegend 63D3 367104 1/100
PE- CD19 BioLegend HIB19 302208 1/100
PE-Siglec-1 BioLegend 7-239 346004 1/100
PEDAZ594-CD69 BioLegend FN50 310941 1/100
PEDAZ594-NKG2D BioLegend 1D11 320828 1/100
PE-Cy5-CD27 Thermofisher O323 15-0279-42 1/100
PE-Cy5-CD25 Biolegend BC96 302608 1/50
Percp-cy5.5-Ki67 (Intracellular) BioLegend Ki-67 350520 1/20
Percp-ef710-gdTCR Thermofisher B1.1 46-9959-42 1/50
Percp-ef710-CD16 Thermofisher CB16 46-0168-42 1/100
PE-Cy7-PD1 BioLegend A17188B 621616 1/100
PE-Cy7-CD141 Biolegend M80 344110 1/100
AF647-CD45 BioLegend HI30 304056 1/100
AF700-CD8 BioLegend HIT8a 300920 1/100
AF700-CD66b BioLegend VI MA81 305114 1/100
APC/Fire750-CD38 BioLegend HB-7 356626 1/100

Validation

All antibodies used have been validated by the manufacturer and used according to the manufacturer's instruction. Antibodies have been re-validated by titrating their concentration. Isotypes and FMO were included in the experiments whenever appropriate.

Clinical data

Policy information about clinical studies
All manuscripts should comply with the ICMJE guidelines for publication of clinical research and a completed CONSORT checklist must be included with all submissions.

Clinical trial registration

N/A: not a clinical trial

Study protocol

N/A: not a clinical trial

Data collection

As described in the Methods. All participants were recruited from the Imperial College Renal and Transplant Centre and its satellite dialysis units, London, United Kingdom, and provided written informed consent prior to participation. Study ethics were reviewed by the UK National Health Service (NHS) Health Research Authority (HRA) and Health and Care Research Wales (HCRW) Research Ethics Committee (reference 20/WA/0123: The impact of COVID-19 on patients with renal disease and immunosuppressed patients). Ethical approval was given. Study volunteers provided informed consent and did not receive financial or other compensation for participating in the study.

We recruited two cohorts of ESKD patients with COVID-19 (Figure 1A). All patients were receiving haemodialysis prior to acquiring COVID-19. The first cohort (Wave 1) were recruited during the initial phase of the COVID-19 pandemic (April-May 2020). Blood samples were taken from 53 patients with COVID-19 (Supplementary Table 1). Serial blood sampling was carried out where feasible (Figure 1B), given the pressure on hospital services and the effects of national lockdown. We also contemporaneously recruited 59 non-infected haemodialysis patients to provide a control group, selected to mirror the age, sex and ethnicity distribution of the COVID-19 cases (Supplementary Figure 1A-C).

The Wave 2 cohort consisted of 17 ESKD patients with COVID-19 infected during the resurgence of cases in January-March 2021 (Supplementary Table 2). These 17 individuals had all been recruited as part of the COVID-19 negative control group during Wave 1, and so a pre-infection sample collected in April/May 2020 (8-9 months preceding infection) was also available. For the Wave 2 cohort, we systematically acquired serial samples for all patients at regular intervals (every 2-3 days over the course of the acute illness) (Figure 1C). Additionally, for 12 of these 17 patients, we acquired convalescent samples at approximately 2 months post the acute COVID-19 episode (range 41-55 days from the initial sample). Convalescent samples were unavailable for four patients who died and for one patient due to logistical difficulties in sample collection.

To minimise variation related to the timing of dialysis, blood samples were taken prior to commencing a haemodialysis session.

Outcomes

As described in the Methods. We assessed disease severity using a four-level ordinal score, categorising into mild, moderate, severe, and critical, based on the WHO clinical management of COVID-19: Interim guidance 27 May 2020. Mild was defined as COVID-19 symptoms but no evidence of pneumonia and no hypoxia. Moderate was defined as symptoms of pneumonia or hypoxia with oxygen saturation (SaO_2) greater than 92% on air, or an oxygen requirement no greater than 4 L/min. Severe was defined as SaO_2 less than 92% on air, or respiratory rate more than 30 per minute, or oxygen requirement more than 4 L/min. Critical was defined as organ dysfunction or shock or need for high dependency or intensive care support (i.e. the need for non-invasive ventilation or intubation). We recorded disease severity scores throughout the illness, such that samples from the same individual could have differing severity scores according to the temporal evolution of the disease. We defined the overall clinical course for each patient as the peak severity score that occurred during the patient's illness. Different downstream analyses utilise either the severity at the time of sample (i.e. the sample-level severity) or the overall clinical course (i.e. the patient-level severity), as described in the relevant sections below.

Flow Cytometry

Plots

Confirm that:

☒ The axis labels state the marker and fluorochrome used (e.g. CD4-FITC).

☒ The axis scales are clearly visible. Include numbers along axes only for bottom left plot of group (a 'group' is an analysis of identical markers).

☒ All plots are contour plots with outliers or pseudocolor plots.

☒ A numerical value for number of cells or percentage (with statistics) is provided.

Methodology

Sample preparation

Cryopreserved PBMCs were thawed in humidified 37°C, 5% CO_2 incubator and resuspended in thawing medium (RPMI, 20% FBS). PBMCs were washed twice with PBS and stained with Zombie Yellow LIVE/DEAD (Biolegend) following the manfacturer's protocol to exclude dead cells. Then, PBMCs were washed twice with FACS buffer (1% BSA, 0.09% Azide, 1 mM EDTA), and Fc receptors were blocked with Human TruStain Fc Receptor Blocking Solution (Biolegend). Then, surface staining were performed using the selected fluorochrome conjugated monoclonal antibodies detailed in Supplementary Table 5 for 20 minutes at 4°C. Following incubation, cells were fixed and permeabilized using the eBioscience™ Foxp3 / Transcription Factor Staining Buffer Set (Invitrogen) for intracellular staining. Cells were incubated with selected antibodies or isotype controls for 30 minutes at 4°C and resuspended in FACs buffer for analysis.

Instrument

Aurora Spectral Flow Cytometry (Cytek®)

Software

FlowJo software, version 10 (Tree Star Inc. Ashland, OR, USA) and Graphpad Prism version 9 were used for analysis of all samples.

Cell population abundance

No FACs sorting were done to PBMCs

Gating strategy

Relevant gating strategies were presented in Supplementary Figure 27 and 28. Prior to gating cell population of interest, cell debris was removed based on FCS/SSC and only live cell (BV570 Zombie Yellow - negative) populations were analyzed.

☒ Tick this box to confirm that a figure exemplifying the gating strategy is provided in the Supplementary Information.
